# Fyn-tau Ablation Modifies PTZ-Induced Seizures and Post-seizure Hallmarks of Early Epileptogenesis

**DOI:** 10.3389/fncel.2020.592374

**Published:** 2020-12-08

**Authors:** Marson Putra, Sreekanth Puttachary, Guanghao Liu, Gloria Lee, Thimmasettappa Thippeswamy

**Affiliations:** ^1^Neuroscience Interdepartmental Program, Iowa State University, Ames, IA, United States; ^2^Department of Biomedical Sciences, College of Veterinary Medicine, Iowa State University, Ames, IA, United States; ^3^Department of Internal Medicine, University of Iowa Carver College of Medicine, Iowa City, IA, United States

**Keywords:** epilepsy, tau, Fyn, seizure, neuroinflammation, neurodegeneration

## Abstract

Both Fyn and tau have been associated with neuronal hyperexcitability and neurotoxicity in many tauopathies, including Alzheimer's disease (AD). Individual genetic ablation of *fyn* or *tau* appears to be protective against aberrant excitatory neuronal activities in AD and epilepsy models. It is, however, still unknown whether ablation of both Fyn and tau can likely elicit more profound anti-seizure and neuroprotective effects. Here, we show the effects of genetic deletion of Fyn and/or tau on seizure severity in response to pentylenetetrazole (PTZ)-induced seizure in mouse models and neurobiological changes 24 h post-seizures. We used Fyn KO (*fyn*^−/−^), tau KO (*tau*^−/−^), double knockout (DKO) (*fyn*^−/−^**/***tau*^−/−^), and wild-type (WT) mice of the same genetic background. Both tau KO and DKO showed a significant increase in latency to convulsive seizures and significantly decreased the severity of seizures post-PTZ. Although Fyn KO did not differ significantly from WT, in response to PTZ, Fyn KO still had 36 ± 8% seizure reduction and a 30% increase in seizure latency compared to WT. Surprisingly, in contrast to WT, Fyn KO mice showed higher mortality in <20 min of seizure induction; these mice had severe hydrocephalous. None of the *tau*^−/−^ and DKO died during the study. In response to PTZ, all KO groups showed a significant reduction in neurodegeneration and gliosis, in contrast to WT, which showed increased neurodegeneration [especially, parvalbumin (PV)-GABAergic interneurons] and gliosis. DKO mice had the most reduced gliosis. Immunohistochemically, phospho-tau (AT8, pS199/S202), Fyn expression, as well as Fyn-tau interaction as measured by PLA increased in WT post-PTZ. Moreover, hippocampal Western blots revealed increased levels of AT8, tyrosine phospho-tau (pY18), and phosphorylated Src tyrosine family kinases (pSFK) in PTZ-treated WT, but not in KO, compared to respective controls. Furthermore, PV interneurons were protected from PTZ-induced seizure effects in all KO mice. The levels of inwardly rectifying potassium (Kir 4.1) channels were also downregulated in astrocytes in the WT post-PTZ, while its levels did not change in KO groups. Overall, our results demonstrated the role of Fyn and tau in seizures and their impact on the mediators of early epileptogenesis in PTZ model.

## Introduction

In 2012, epilepsy was reported to have affected ~50 million people worldwide (Hesdorffer and Begley, 2013). The progression of seizures in epilepsy has been attributed to diverse molecular mechanisms including mechanisms that involve tau and Fyn (Ittner et al., 2010; Sharma et al., 2018). This association was based on evidence that epileptic seizures and cognitive deficits are frequently reported in patients with Alzheimer's Disease (AD), the most common form of tauopathies (Amatniek et al., 2006; Palop and Mucke, 2009). Analysis of surgically resected epileptic brains revealed similar pathologies to those observed in AD including abnormally phosphorylated tau and increased amyloid precursor protein (APP) (Thom et al., 2011; Tai et al., 2016; Smith et al., 2019; Gourmaud et al., 2020). In addition to tau pathology, increased Fyn expression was evident in AD brains (Shirazi and Wood, 1993; Ho et al., 2005). Although there is no study yet demonstrating Fyn expression in epileptic brains, several animal studies have linked the contribution of Fyn to neuronal hyperexcitability and seizure manifestation (Chin et al., 2004; Palop et al., 2007; Sharma et al., 2018). Moreover, AD patients and animal models of AD (Roberson et al., 2011; Vossel et al., 2017) exhibit seizure properties as disease progresses, suggesting the plausible involvement of Fyn-tau interaction in the development of seizures. Therefore, tau and Fyn could be promising therapeutic targets to combat seizures and seizure-associated pathologies.

Tau, a microtubule-associated protein, is mainly found in neurons with some expression in glia (LoPresti et al., 1995). Physiologically, tau is highly involved in cytoskeletal stability, axonal development, and intracellular trafficking (Dixit et al., 2008; Wang and Mandelkow, 2016). On the other hand, Fyn, a member of the Src family tyrosine kinases (SFKs), regulates the development of the central nervous system, synaptic plasticity, and maturation of the peripheral immune system (Grant et al., 1992; Salmond et al., 2009). Several studies have shown evidence of Fyn-tau interaction resulting in tyrosine phosphorylation of tau by Fyn on Tyr-18 (Lee et al., 1998, 2004; Bhaskar et al., 2005; Ittner et al., 2010; Rush et al., 2019). Tau is needed for Fyn to be localized to dendritic spines (Xia and Götz, 2014; Li and Götz, 2017). These interactions begin with tau phosphorylation at Tyr18 by Fyn, enabling localization of Fyn to dendritic spines. In the postsynaptic dendritic spine, Fyn-tau complexes then associate with postsynaptic density-95 (PSD-95), where N-methyl-D-aspartate receptor (NMDAR) on subunit NR2B at Tyr1472 is phosphorylated by Fyn. As a result, these PSD-95/tau/Fyn complexes allow the opening of NMDAR, enhancing the influx of Ca^2+^, which then results in hyperexcitation and neurotoxicity (Ittner et al., 2010; Ittner and Götz, 2011). A modification of tau on the Fyn binding site or pharmacological inhibition of this interaction prevents complex formation resulting in the reduction of seizure and neuronal excitotoxicity in experimental AD models (Ittner et al., 2010; Rush et al., 2019).

A reduction in tau protein lessens seizure severity in various epilepsy models, ranging from pharmacologically induced seizure (DeVos et al., 2013; Li et al., 2014) to genetic models (Holth et al., 2013; Gheyara et al., 2014). In addition, microgliosis appears to be aggravated by the hyperphosphorylation of tau, suggesting a pivotal role for tau pathology in mediating neuroinflammation (Bhaskar et al., 2010). Furthermore, cognitive deficits were improved, and mortality was reduced by depleting tau in AD mice (Roberson et al., 2007). In relation to Fyn, genetic ablation of tau downregulated Fyn activity (Ittner et al., 2010; Liu et al., 2019) while Fyn depletion or inhibition also decreased abnormal tau phosphorylation (Kaufman et al., 2015; Liu et al., 2020; Tang et al., 2020). On the other hand, Fyn depletion attenuated seizure development in a mouse model of temporal lobe epilepsy (Chun et al., 2004; Sharma et al., 2018) while its overexpression enhanced seizure susceptibility (Cain et al., 1995; Kojima et al., 1998) as well as tau aggregation (Xia and Götz, 2014; Li and Götz, 2017). Past studies have demonstrated reduced glial-mediated neuroinflammation with pharmacological Fyn inhibition in kainic acid (KA)-induced epilepsy (Sharma et al., 2018), AD or tauopathy (Kaufman et al., 2015; Tang et al., 2020), and Parkinson's disease (Panicker et al., 2019) models. In support of these observations, mice with overexpression of Fyn displayed high mortality, hyperactivity, and significant weight loss compared to WT (Xia and Götz, 2014). These studies collectively underscore the roles of Fyn and tau in the regulation of synaptic and neuronal functions as well as the inflammatory state of the brain.

Neuroinflammation and neurodegeneration are the most prominent pathogenic features of epilepsy. Microgliosis and astrogliosis are evident in tau and Fyn associated disorders (Kaufman et al., 2015; Liu et al., 2020). Reactive astrocytes fail to uptake glutamate and K^+^ clearance leading to increased epileptiform discharge (Devinsky et al., 2013). Upregulation of Fyn mRNA is attributable to astrocyte activation in the KA-induced epilepsy mouse model (Chun et al., 2004) and in lipopolysaccharide (LPS)-stimulated primary neuronal cultures (Lee et al., 2017). Tau phosphorylation also increased reactive astrocytes expression (Forman et al., 2005; Liu et al., 2020). Reactive glia can mediate neuronal death (Barres, 2008). In a mouse AD model that was overexpressing Fyn, tau KO protected calbindin GABAergic interneurons (Palop et al., 2007; Roberson et al., 2011). Despite these studies demonstrating the roles of Fyn and tau in neuroinflammation and neurodegeneration, the mechanism by which Fyn and tau modulate glial activation and induce neuronal loss at the cellular level in seizure models remain unknown.

To understand the effects of combined action of Fyn and tau in pathologies associated with seizures, in this study, we used gene KO mice; *fyn*^−/−^, *tau*^−/−^, and *fyn*^−/−^/*tau*^−/−^ double KO. We hypothesized that the combined absence of Fyn and tau would mitigate the severity of seizures, gliosis, and neurodegeneration at 24 h following a chemoconvulsant injection. To investigate gene KO effects on behavioral seizures, the mice were injected with pentylenetetrazol (PTZ), and 24 h later, neurobiological changes were analyzed. Behavioral seizures were significantly reduced in tau KO and DKO relative to WT post-PTZ despite there was no difference between DKO and tau KO mice. Fyn KO mice, however, only showed a 30–40% seizure reduction compared to WT mice. Molecularly, ablating both Fyn and tau individually or in combination ameliorated seizure-associated brain pathologies such as gliosis, neurodegeneration, and loss of GABAergic interneurons. Although DKO had nearly similar effects on seizure-associated pathologies relative to single Fyn and tau KO, DKO revealed a higher impact on mitigating microgliosis. Our results highlight the involvement of Fyn and tau in the early phase of epileptogenesis supporting our hypothesis of the pro-epileptogenic roles of Fyn and tau.

## Materials and Methods

### Animal Source and Care

**Figure 1:**
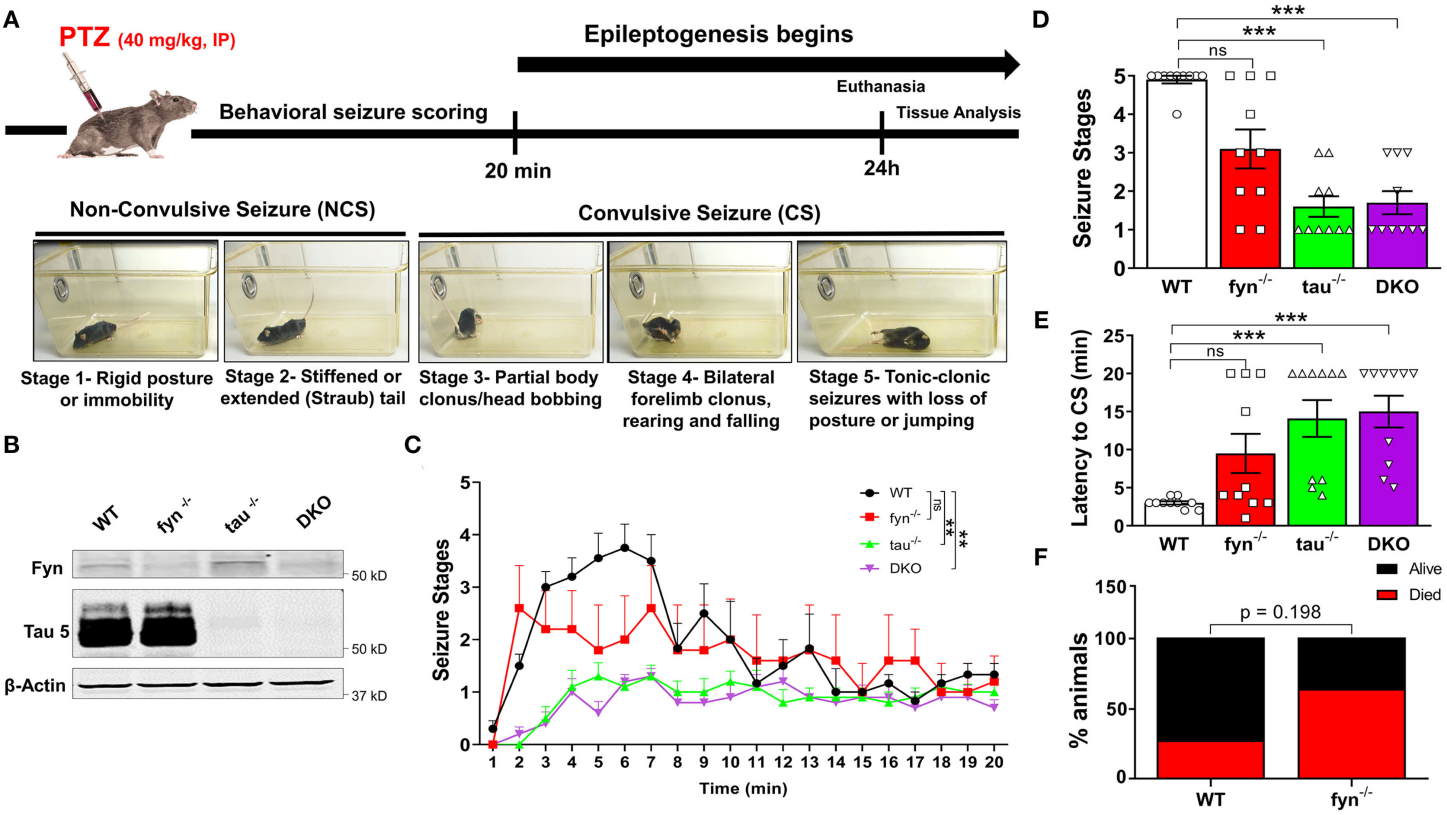
The experimental design, genotype validation, and seizure response to PTZ. **(A)** Timeline showing the experimental design and representative images of behavioral seizure types in response to PTZ. Behavioral seizures scoring was conducted for 20 min after PTZ injection. All mice were sacrificed at 24 h post-PTZ and tissues harvested for analysis. **(B)** Representative WB of brain lysate of mice used in the study to confirm genotype. **(C)** Time course of behavioral SE during the initial 20 min following PTZ administration. Both *tau*^−/−^ and DKO mice had significantly less CS compared to WT, while *fyn*^−/−^ was not significantly different from WT littermates (*n* = 7–10 per group; **p < 0.01; ns, not significant; repeated two-way ANOVA with Tukey's multiple *post-hoc* test). **(D)** The seizure severity was significantly decreased in *tau*^−/−^ and DKO, while *fyn*^−/−^ only had a 30% reduction, which did not significantly differ from WT (*n* = 7–10; ***p < 0.001, ns, not significant; non-parametric Kruskal-Wallis with Dunn's *post-hoc* test). **(E)** Latency to the onset of CS was significantly increased in *tau*^−/−^ and DKO. Although *fyn*^−/−^ mice showed a trend of increased seizures latency, no significant difference was observed compared to WT (*n* = 7–10; ***p < 0.001, ns, not significant; non-parametric Kruskal-Wallis with Dunn's *post-hoc* test). **(F)** Relative to WT, *fyn*^−/−^ mice had an unexpected higher mortality mouse in response to PTZ. However, the difference was not significant (*n* = 15, *p* = 0.198, Fischer's exact test). Data presented as mean ± SEM.

Homozygous *fyn*^−/−^ mice bred on S129 × C57BL/6J hybrid strain background and *tau*^−/−^ on C57BL/6J background used in this study were obtained from Jackson laboratory. Wildtype (WT) and *fyn*^−/−^/*tau*^−/−^ mice were generated as previously described (Liu et al., 2019). To assess hydrocephalus, mice were screened by MRI. We used a cohort of 96 male and female mice (12 weeks old) in this study; *fyn*^−/−^/*tau*^−/−^ DKO (20 males, four females), *fyn*^−/−^ (10 males, 14 females), and *tau*^−/−^ (14 males, nine females), and WT (19 males, six females). The 12 mice that died (4 WT, 8 Fyn KO) during the experiment were excluded from the analyses. The WT and vehicle-treated KO mice served as control. Western blots were routinely performed to confirm genotype (Figure 1B). All mice were housed on a regular cycle of 12 h light/ 12 h dark at 22°C with free food and water access at the University of Iowa. All *in vivo* experiments were conducted at the University of Iowa as per the approved protocols by the Institutional Animal Care and Use Committee (IACUC). Biochemical experiments were carried out at Iowa State University.

### Pentylenetetrazole-Induced Seizures and Behavioral Seizure Scoring

GABA_A_ receptor antagonist pentylenetetrazol (PTZ) was prepared sterile in phosphate-buffered saline (PBS) at a concentration of 4 mg/ml and injected intraperitoneally at a dose of 40 mg/kg to induce seizures. After PTZ injection, all mice were placed in a transparent cage for 20 min for behavioral seizure scoring and video recording for secondary validation and seizure quantification (Liu et al., 2019). The experimenters were blinded to the genotypes of the mice when analyzing the videotape/direct observation and quantification of the seizure severity was based on published scoring criteria (Borges et al., 2003). The experimental design is illustrated in Figure 1A. The modified version of the Racine scale was used as follows: 0, normal activity; 1, rigid posture or immobility; 2, stiffened or tail extension; 3, rearing with facial and manual automatism a well as partial body clonus, including forelimb clonus; 4, rearing and falling; 5, tonic-clonic seizures with loss of posture or bouncing (Figure 1A). Stage 1 and 2 were classified as non-convulsive seizures, whereas stages 3–5 were convulsive seizures (Racine, 1972; Tse et al., 2014).

### Tissue Processing and Immunohistochemistry

The mice assigned for immunohistochemistry (IHC) were euthanized at 24 h post-PTZ by an overdose of pentobarbital (100 mg/kg, i.p.). Trans-cardiac perfusion with 4% paraformaldehyde (PFA) in PBS was used to fix the tissues as previously described (Putra et al., 2020b). Briefly, the brain was harvested, post-fixed overnight in 4% PFA, cryopreserved in 20% sucrose, and stored at 4°C overnight. Brain tissue was then incubated in gelatin embedding-solution (PBS containing 0.1% sodium azide, 7.5% sucrose, and 15% porcine gelatin). The tissue blocks were snap-frozen with liquid nitrogen-cooled isopentane, immediately stored at−80°C. Frozen tissue blocks were then sectioned coronally (16 μm) on a cryostat (Cryostar NX70, ThermoScientific, MA, USA). Collected brain slices were mounted sequentially onto chrome-alum-gelatin pre-coated glass slides (four sections/slide, 225 μm apart for mouse brain) as described previously (Puttachary et al., 2016a). This sampling method yielded representative rostral-caudal aspects of the brain on a single slide. The slides were then stored at 20°C until processed for IHC.

Brain sections were subjected to antigen retrieval by immersion in pre-heated citrate buffer (10 mM citric acid, 0.05% Tween 20, and pH 6) to 90°C for 20 min prior to IHC. Sections were then washed in PBS a few times to remove PFA traces and antigen retrieval reagents, blocked with PBS containing 10% donkey serum and 0.02% Triton-X100 for 1 h at room temperature. Double or triple immunolabeling was performed on control groups, and PTZ treated groups using the same reagents and antibodies. Afterward, primary antibodies diluted in PBS containing 2.5% donkey serum, 0.25% sodium azide, and 0.1% Triton X-100 were added to the sections for 16–18 h incubation at 4°C. Sections were rinsed with PBS and incubated with either dye-conjugated or biotinylated secondary antibody at room temperature for 1 h. After washing with PBS, incubated with biotinylated secondary antibodies, sections were treated with dye-streptavidin for 45 min and then thoroughly rinsed in PBS. Sections were then treated with a nuclear-stain, 4′,6-diamidino-2- phenylindole (DAPI) and cover-slipped. Negative control was the section without primary antibody staining, while positive control was a section from a known SE-induced brain pathology stained with both primary and secondary antibodies (e.g., thalamus for IBA1). The list of reagents and antibodies used in the study are shown in Table 1.

**Table 1:** Antibodies and reagents used in the experiments.

**Antibodies**	**Source**	**Dilution**	**Identifier**	**Application**
Goat Anti-IBA1	Abcam	1:300	ab5076	IHC
Mouse Anti-Glial Fibrillary Acidic Protein	Millipore	1:400	AB5804	IHC
Rabbit Anti-NeuN	Millipore	1:200	MAB377	IHC
Rabbit Anti-Fyn	Santa-Cruz	1:100	sc-16	IHC, WB, PLA
Mouse Anti-Fyn	Thermoscientific	1:1000	MA1-19331	WB
Rabbit Anti-Phospho-SFK (Tyr416)	Cell signaling	1:1000	CST-2101	WB
Mouse Anti-Phospho-tau (AT8) (pSer199/Ser202/Thr205)	Thermoscientific	1:300	MN-1020	IHC
Mouse Anti-Phospho-tau (pY18) (Tyr18)	Gloria Lee	1:1000	Gift (Lee et al., 2004)	WB
Mouse Anti-Tau (DA9)	Peter Davies	1:50	Gift (RRID:AB_2716723)	PLA
Mouse Anti-Tau5	Late Lester I. Binder	1:1000	Gift (RRID:AB_2721194)	WB
Rabbit Anti-Kir4.1	Santa-Cruz	1:100	sc-23637	IHC, WB
FlouroJade B	Histo-Chem Inc	1:1000	FJB	IHC
Biotin-SP-AffiniPure Donkey Anti-Goat IgG (H+L)	Jackson ImmunoResearch	1:300	705-065-147	IHC
Biotin-SP-AffiniPure Donkey Anti-Mouse IgG (H+L)	Jackson ImmunoResearch	1:300	715-065-150	IHC
Biotin-SP-AffiniPure Donkey Anti-Rabbit IgG (H+L)	Jackson ImmunoResearch	1:300	711-065-152	IHC
Cy3™ Streptavidin	Jackson ImmunoResearch	1:300	016-160-084	IHC
Fluorescein (FITC)-AffiniPure Donkey Anti Rabbit IgG (H+L)	Jackson ImmunoResearch	1:300	711-095-152	IHC
Cy3-AffiniPure Donkey Anti-Rabbit IgG (H+L)	Jackson ImmunoResearch	1:300	711-165-152	IHC
β-Actin Mouse or Rabbit	Sigma Aldrich	1:10,000	A5316	WB
IRDye® 800CW Donkey anti-Rabbit or Mouse IgG (H + L)	LI-COR Biosciences	1:10,000	926-32213	WB
IRDye® 680LT Donkey anti-Rabbit or Mouse IgG (H + L)	LI-COR Biosciences	1:10,000	926-68022	WB
**Reagents or drugs**	**Source**	**Identifier**		
Pentylenetetrazole (PTZ)	Sigma-Aldrich	P6500		
Duolink Insitu PLA probe anti-rabbit PLUS	Sigma-Aldrich	DUO92002		
Duolink Insitu PLA probe anti-mouse MINUS	Sigma-Aldrich	DUO92004		
Duolink Insitu Detection Reagent Orange	Sigma-Aldrich	DUO92007		
Donkey Normal Serum	Abcam	ab7475		

### Flouro-Jade B Staining

Degenerating neurons in the hippocampus and entorhinal cortex (ENT) were identified using NeuN and Flour-Jade B (FJB) double staining. The sections were processed for NeuN immunolabeling as described above. Afterward, FJB staining was performed using the modified protocol from our previously published studies (Puttachary et al., 2016a; Putra et al., 2020b). Sections were then immersed in the following order: 3 min in 100% ethanol, 3 min in 70% ethanol, 1 min in distilled water. Sections were oxidized in 0.006% solution of potassium permanganate (KMnO_4_) for 10 min on a shaker. After 3–4 rinses in distilled water for 1 min, sections were transferred to 0.1% acetic acid containing a 0.0003% solution of FJB for 10 min with gentle shaking in a dark room. Sections were rinsed with distilled water three times, air-dried for 3 h, cleared with xylene, and mounted with a hard-mounting medium, Acrytol (Surgipath, Leica Biosystems, IL).

### Proximity Ligation Assay

Slides containing brain sections from each group for PLA analysis were processed similar to the IHC method up through the primary antibodies incubation step as described above. Following antigen retrieval and blocking steps, the sections were incubated with primary antibodies of anti-rabbit Fyn and anti-mouse total tau (DA9). Sections were also incubated with anti-chicken MAP2, a dendritic marker, in order to visualize the distribution of Fyn-tau complexes within dendritic compartments. The PLA kit was purchased from Sigma (Duolink® *insitu*), and assay protocol was based on the manufacturer's instructions with a modification for tissue slices (Gomes et al., 2016). Once incubation with primary antibodies was complete, the sections were rinsed with Buffer A, and later incubated with PLA probes Rabbit-plus and Mouse-minus diluted in Duolink® antibody diluent for 1 h at 37°C. Upon completion, the sections were rewashed with Buffer A, followed by incubation with DNA ligation reagents for 30 min at 37°C. After another wash with Buffer A, enzymatic amplification and PLA hybridization were performed by adding the corresponding reagents onto the sections for 100 min at 37°C. Section were then washed in a dark room with Buffer B to prevent signal bleaching from light. Following the completion of PLA, the sections were incubated with an anti-chicken dye-conjugated secondary antibody for MAP2 for 1 h at room temperature. The sections were washed with PBS and coverslipped water-based mounting medium. The details on reagents and antibodies for this experiment are shown in Table 1.

**Figure 2:**
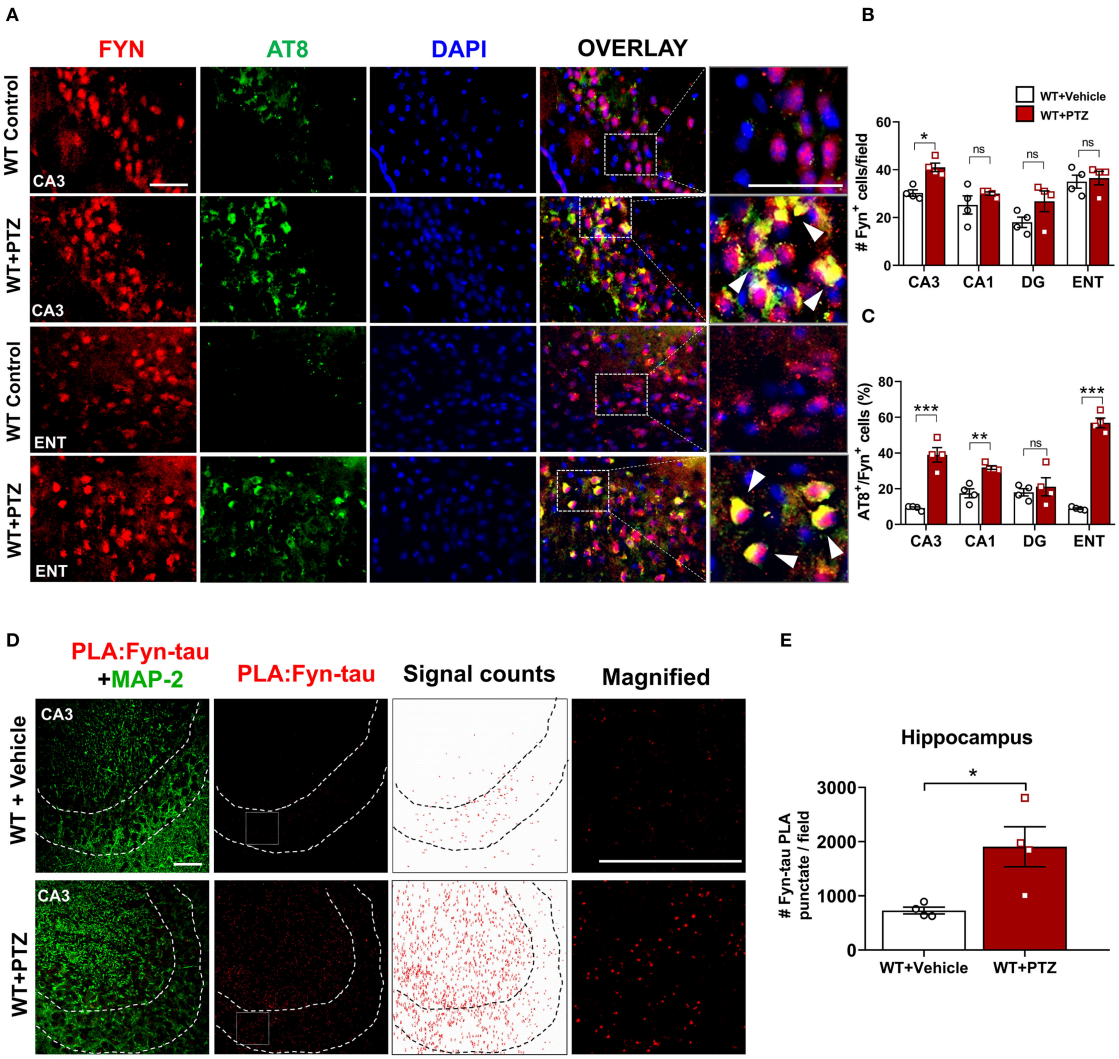
Increased expression of Fyn, AT8 (phospho-tau), and Fyn-tau complexes after PTZ-induced seizures in the WT mice. **(A)** Double immunostaining of AT8 (green) and Fyn (red) in WT mice at 24 h post-PTZ showed a significant increase of AT8+Fyn double-labeled cells (yellow) in the CA3 of the hippocampus and ENT (representative images are shown). Arrowheads indicate the co-localization of AT8 with Fyn in neurons. Sections were counterstained with DAPI (blue). Scale bar, 50 μm. **(B)** Fyn immunopositive cell quantification in the hippocampus (CA1, CA3, and DG) and ENT. A significant increase of Fyn expressing cells was observed in CA3 region. **(C)** AT8/Fyn double labeled cell quantification in the hippocampus and ENT. In response to PTZ, AT8 expression was significantly upregulated in CA3, CA1, and ENT but not in DG (*n* = 4–5; 4 sections/animal; *p < 0.05, **p < 0.01, ***p < 0.001, ns, not significant; repeated measure two-way ANOVA with Sidak's *post-hoc* for multiple comparisons). **(D)** Fyn-tau complexes, detected by PLA (red), and MAP2 labeling (green) were observed in the vehicle, and PTZ treated WT mice, showing an increase of Fyn-tau PLA punctates in CA3 (magnified photograph). Scale bar 100 μm. **(E)** Quantitative analysis of Fyn-tau PLA punctates showed a significant increase of PLA complexes in the hippocampus of WT+PTZ as compared to WT+Vehicle (*n* = 4, 4 sections/animal; *p < 0.05; unpaired Student's *t*-test). Data presented as mean ± SEM.

For Fyn-tau interaction analysis, the sections were visualized under a confocal microscope (Nikon Ti Eclipse) with 40x objective, and images were acquired in z-stack focus. Ten random z-stacked images with 1 μm intervals were collected from CA1, CA3, and DG regions of both hippocampi from at least three sections for each animal. The images were then processed for maximum projection allowing the maximum visibility of the PLA punctate and later analyzed with *ImageJ* (Figure 2D) and quantified as described in the literature (Gomes et al., 2016). The number of punctate counts per field was then plotted for each group. To validate the PLA signals, negative controls where either one of the antibodies for interaction (Fyn or tau) was not added, or a section from one of the KO mice were used. A validation of Fyn-tau PLA signals from each genotype is shown in Supplementary Figure 1.

### Microscopy and Cell Quantification

Immunostained sections were visualized using a fluorescence microscope (Zeiss Axiovert 200) and processed with HCImage live (version 4.1.2.) software as previously described (Putra et al., 2020b). Briefly, the photographs were taken at multiple magnifications (10x, 20x, and 40x) with consistent exposure time. *ImageJ* was used to determine the counting area (mm^2^) and blind manual cell counts. A bilateral quantitation of immunopositive cells with a visible nucleus (DAPI^+^) was undertaken from a minimum of four sections per animal involving rostral to caudal aspects of the hippocampus and ENT as previously described (Puttachary et al., 2016a). To avoid over-counting bias resulting from consideration of cell fractions as whole cells, Abercrombie correction was applied (Abercrombie and Johnson, 1946) as previously described (Putra et al., 2020a). Furthermore, index change of the pathologies such as microgliosis, astrogliosis, neurodegeneration was determined by subtracting the cell count in WT + PTZ from cell count in each KO + PTZ then dividing the result with cell count in WT + PTZ. The values deviating from 0 indicates the degree of change in corresponding pathology relative to WT. This analysis was performed to compare the overall neuroprotective effects of each KO in all regions globally.

### Skeleton Analysis of Microglia

A microglial morphometric analysis was carried out using the skeletonization method to quantify microglia morphology in IHC images of the hippocampus. A modified method was adopted from the published studies (Harvey et al., 2015; Morrison et al., 2017). The images of IBA1^+^ sections were captured on a 40x lens using Zeiss Axiovert 200. A series of images with multiple focuses photographed on the same field was stacked manually (10 images in total) using the “Stack Focuser” plugin (http://imagejdocu.tudor.lu/) to create a single image with enhanced visualization of microglia processes. Images were randomly sampled from rostral to caudal regions of the hippocampus. Afterward, acquired images were loaded onto *ImageJ* software, despeckled to eliminate background noise. Fluorescence intensity in each image was kept consistent to minimize the variability of measurement between samples. The cell body area of each microglia in a given image was determined after converting the pixel into micrometer (μm) scale. The image was then binarized and skeletonized using *ImageJ* software. The Analyze Skeleton plugin (http://imagejdocu.tudor.lu/) was used to obtain data on the number of endpoints and the longest processes path per cell on the frame. Approximately, 50–70 randomly selected microglia cells in the hippocampus were analyzed in each group.

### Western Blotting

Hippocampal tissue lysates were prepared on wet ice (4°C) by homogenizing the tissue in a cocktail of RIPA buffer and protease and phosphatase inhibitors (Thermo-Scientific, USA). Protein normalization of the lysates was performed by employing the Bradford protein assay, as previously described (Putra et al., 2020b). Each sample with the equivalent amount of hippocampal tissue proteins was resolved on 8 or 10% SDS-PAGE. Following protein transfer to a nitrocellulose membrane, the membrane was then washed several times in wash buffers [0.1% Tween in PBS (PBST)], then blocked for 1 h at 25°C with a blocking buffer for fluorescent Western blotting (Rockland Immunochemicals, USA). The incubation of the membrane with primary antibodies was carried out for 16 h at 4°C. After several washes, an infrared dye-tagged secondary antibody, either 680 or 800 nm, was applied for 1 h. Odyssey IR imaging system (LiCor, USA) was used to view the binding of antibodies. *ImageJ* software was used to analyze the target protein across the groups by normalizing the target protein band intensity to the β-actin band intensity from the same sample. The full blot images are available in Supplementary Figure 2.

### Methodological Rigor and Statistical Analyses

All animals were randomized and coded before the treatment protocols began. All experimenters were blinded to the genotype of the mice and treatment groups until the data analyses were entirely analyzed. Animals that died during behavioral scoring were excluded from the study. Statistical analyses were done in Prism 8.0 (GraphPad Software) and R-Studio version 1.1.463. The normality of each dataset was evaluated with the Shapiro-Wilk test, and outliers were detected with Robust Regression and Outlier Removal Test (ROUT). When comparing multiple groups, the significance of normal data was detected using one-way ANOVA with Tukey's *post-hoc* test, whereas non-normal data were evaluated with the Kruskal-Wallis test with Dunn's *post-hoc* test. Data within two factors were analyzed with two-way ANOVA with Tukey's or Sidak's multiple comparisons. Data collected over time or quantified from the same brain specimens were analyzed with a mixed-effect model with Tukey's *post-hoc*. A paired comparison between two groups was achieved with unpaired Student's *t*-test for normal data and the Mann-Whitney test for non-normal data. The mortality was analyzed using Fisher's Exact test. The detail of all statistical tests applied in this study is shown in Table 2. All data were presented as mean and standard error mean (±SEM). The graphs of mixed-effect analysis were plotted as group median with 95% of confidence interval. Differences between groups with *p* < 0.05 were considered statistically significant.

**Table 2:** Details of statistical analysis applied for each experiment/figure.

**Figure**	**Panel**	**Test**
1	C	RM-two-way ANOVA: *F*_(2,31)_ = 7.112; *p* = 0.0009; Tukey's *post-hoc*;
	D	Non-parametric Kruskal-Wallis test; *p* < 0.0001; Dunn's *post-hoc*. **WTvs fyn**^**−/−**^ (*p* = 0.1547), **WT** vs. **tau**^**−/−**^ (*p*-0.0001), **WT** vs. **DKO** (*p* = 0.0002).
	E	Non-parametric Kruskal-Wallis test; *p* < 0.0001; Dunn's *post-hoc*. WTvs fyn^−/−^ (*p* = 0.1970), WT vs. tau^−/−^ (*p*-0.0008), WT vs. DKO (*p* = 0.0003).
	F	Fisher's exact test, two-sided; *p* = 0.1984
2	B	RM-two-way ANOVA: *F*_(1,6)_ = 9.746; *p* = 0.0205; Sidak's *post-hoc*;
	C	RM-two-way ANOVA: *F*_(1,6)_ = 147.6; *p* < 0.0001; Sidak's *post-hoc*;
	E	Unpaired *t*-test, two-tailed df = 6, *t* = 3.147; *p* = 0.0199
3	B	two-way ANOVA: *F*_(1,13)_ = 8.046; *p* = 0.0140; Tukey's *post-hoc*;
	C	two-way ANOVA: *F*_(1,13)_ = 9.458; *p* = 0.0089; Tukey's *post-hoc*;
	D	two-way ANOVA: *F*_(1,25)_ = 11.56; *p* = 0.0023; Tukey's *post-hoc*;
	E	two-way ANOVA: *F*_(1,13)_ = 11.56; *p* = 0.6425; Tukey's *post-hoc*;
	F	two-way ANOVA: *F*_(1,13)_ = 0.01729; *p* = 0.8974; Tukey's *post-hoc*;
4	B	two-way ANOVA; Tukey's *post-hoc*; **CA3** [*F*_(1,25)_ = 62.66]; **CA1** [*F*_(1,25)_ = 84.62], **DG** [*F*_(1,25)_ = 47.18], **ENT** [*F*_(1,24)_ = 374.8].
	C	Mixed-effects model: *F*_(2,36)_ = 10.52; *p* = 0.0003; Tukey's *post-hoc*. **DKO** vs. **fyn**^**−/−**^ (*p* = 0.0216), **DKO** vs. **tau**^**−/−**^ (*p* = 0.0282), **fyn**^**−/−**^ vs. **tau**^**−/−**^ (*p* = 0.9956).
5	B	two-way ANOVA: *F*_(1,26)_ = 12.28; *p* = 0.0017; Tukey's *post-hoc*;
	C	two-way ANOVA: *F*_(1,26)_ = 4.289; *p* = 0.0484; Tukey's *post-hoc*;
	D	two-way ANOVA: *F*_(1,26)_ = 51.14; *p* < 0.0001; Tukey's *post-hoc*;
6	B	two-way ANOVA; Tukey's *post-hoc*; **CA3** [*F*_(1,25)_ = 31.35]; **CA1** [*F*_(1,24)_ = 28,56], **DG** [*F*_(1,25)_ = 36.43], **ENT** [*F*_(1,24)_ = 53.91].
	C	Mixed-effects model: *F*_(2,9)_ = 4.339; *p* = 0.0479; Tukey's *post-hoc*. **DKO** vs. **fyn**^**−/−**^ (*p* = 0.1250), **DKO** vs. **tau**^**−/−**^ (*p* = 0.0496), **fyn**^**−/−**^ vs. **tau**^**−/−**^ (*p* = 0.8236).
7	B	two-way ANOVA; Tukey's *post-hoc*; **CA3** [*F*_(1,24)_ = 92.23]; **CA1** [*F*_(1,24)_ = 80.96], **DG** [*F*_(1,24)_ = 84.41], **ENT** [*F*_(1,24)_ = 55.52].
	D	two-way ANOVA: *F*_(1,24)_ = 11.83; *p* = 0.0021; Tukey's *post-hoc*;
8	B	two-way ANOVA; Tukey's *post-hoc*; **CA3** [*F*_(1,24)_ = 59.23]; **CA1** [*F*_(1,24)_ = 38.30], **DG** [*F*_(1,24)_ = 220.2], **ENT** [*F*_(1,24)_ = 61.42].
	C	Mixed-effects model: *F*_(2,9)_ = 0.7923; *p* = 0.4820; Tukey's *post-hoc*. **DKO** vs. **fyn**^**−/−**^ (*p* = 0.1573), **DKO** vs. **tau**^**−/−**^ (*p* = 0.7982), **fyn**^**−/−**^ vs. **tau**^**−/−**^ (*p* = 0.5660).
9	B	two-way ANOVA; Tukey's *post-hoc*; **CA3** [*F*_(1,24)_ = 10.71]; **CA1** [*F*_(1,24)_ = 8,283], **DG** [*F*_(1,25)_ = 0.016], **ENT** [*F*_(1,26)_ = 9.332].
	C	Mixed-effects model: *F*_(2,36)_ = 2.919; *p* = 0.4820; Tukey's *post-hoc*. **DKO vs fyn**^**−/−**^ (*p* = 0.0925), **DKO** vs. **tau**^**−/−**^ (*p* = 0.1219), **fyn**^**−/−**^ vs. **tau**^**−/−**^ (*p* = 0.9896).

*N numbers are provided in the figure legends*.

## Results

### Fyn and Tau KO Mice Demonstrated Reduced Severity of Seizures After PTZ Injection

To evaluate the anti-seizure effects of DKO in comparison with Fyn KO, tau KO, and WT, we injected PTZ intraperitoneally to the mice and blindly scored their behavioral seizures. Following PTZ administration, WT mice reached convulsive seizures (CS) within 2 min. In contrast, tau KO mice and DKO had a significantly lower number of CS over time, while Fyn KO mice were not different from WT (Figure 1C). Cumulative seizure severity score showed a significant reduction in severity in tau KO and DKO compared with WT. Although Fyn KO mice also had a 36 ± 8% reduction in seizure severity compared to WT, it was not statistically significant (Figure 1D). Surprisingly, Fyn KO mice were more susceptible to PTZ-induced death, with 63.64% mortality, while WT mice accounted for 27.37% death (Figure 1F). Moreover, both tau KO and DKO mice had significantly higher latency to CS compared to WT mice (Figure 1E). Fyn KO mice only showed 30% higher latency than those in WT mice, which was not significantly different from the WT control (Figure 1E). Fyn KO and tau KO mice also showed no significant differences in seizure severity and latency. Moreover, DKO mice had a similar seizure activity as tau KO mice.

### Increased Fyn Expression, Hyperphosphorylated Tau, and Fyn-tau Complexes Following 24 h PTZ Injection in WT

Increased hyperphosphorylated tau is evident in KA-induced TLE and observed as early as 1 h following KA (Liang et al., 2009; Alves et al., 2019). Likewise, Fyn activation was elevated as early as 4 h post-KA and persisted for several weeks in the mouse KA model of epilepsy (Sharma et al., 2018). To investigate the degree of tau pathology and Fyn expression in WT following PTZ, we performed immunofluorescence on brain sections to examine phospho-tau [AT8 (pS199/S202)] and Fyn. Representative microscopic images showed that AT8 co-localized with Fyn in CA3 of the hippocampus and ENT (Figure 2A). Upon PTZ injection, at 24 h, Fyn expression was significantly upregulated in the CA3 of the hippocampus. Although Fyn expression did not significantly change in CA1 and Dentate Gyrus (DG), there was a trend of increase in those regions following PTZ administration compared to WT control. In ENT, however, there was no difference in Fyn expression between WT+PTZ and WT control (Figure 2B). On the other hand, the percentage of AT8/Fyn co-expressing cells was significantly increased in the CA1 and CA3 of the hippocampus and ENT. There was no significant difference in DG between groups (Figure 2C). Furthermore, PLA analysis showed a significant increase of PLA signals (number of puncta per field) for Fyn-tau complexes in the hippocampus at 24 h post-PTZ (Figures 2D,E). Overall, these results demonstrated that acute seizure induction with PTZ during the first 24 h increases phospho-tau, Fyn expression, and Fyn-tau interaction.

### Fyn and Tau Ablation Attenuated Seizure-Induced Hyperphosphorylated Tau and Fyn Activation

**Figure 3:**
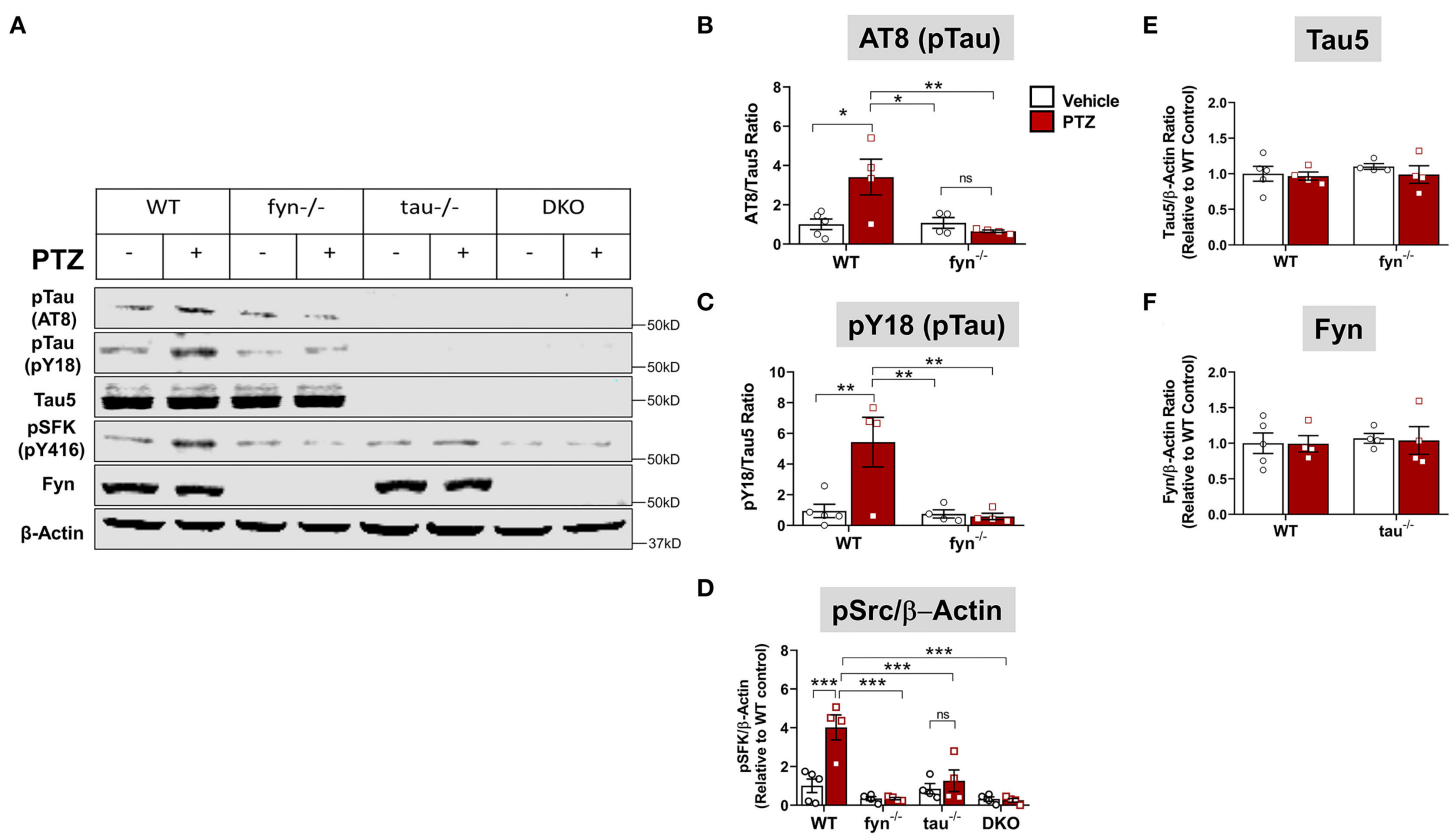
Fyn or tau or both KO attenuated PTZ-induced abnormal tau hyperphosphorylation and Fyn activation. **(A)** Representative hippocampal WB images showed increased tau hyperphosphorylation at AT8 and pY18 epitopes, and activated Fyn (measured by pSFK) in WT+PTZ mice, but not in any KOs. **(B,C)** Quantification of AT8 and pY18 normalized to total tau (Tau5). Compared to WT+PTZ, Fyn KO+PTZ mice showed a significant decrease in tau hyperphosphorylation at AT8 and Y18. **(D)** Quantification of pSFK normalized to β-Actin showed a significant increase in pSFK in WT+PTZ mice relative to WT+Vehicle mice, while all KOs significantly decreased their levels. **(E,F)** Quantification of total tau (Tau 5) or Fyn normalized to β-Actin showed no differences between groups (*n* = 4–5; ***p < 0.001, **p < 0.01, *p < 0.05, ns, not significant; two-way ANOVA with Tukey's *post-hoc* test for multiple comparisons). Data presented as a mean ± SEM.

To further determine tau pathology and Fyn activation in KO mice following PTZ-induced seizures at 24 h, we performed Western blots from whole hippocampal lysates (Figure 3A). We found that WT+PTZ mice showed significantly increased hyperphosphorylated tau markers, AT8 and pY18, by 3.5-fold and 6-fold, respectively, compared to WT mice treated with vehicle (Figures 3B,C). Fyn KO mice treated with PTZ have significantly reduced AT8 and pY18 compared to WT+PTZ mice while there was no difference in AT8 and pY18 expression between Fyn KO mice treated with PTZ and the vehicle (Figures 3B,C). As expected, all tau proteins were absent in both tau KO and DKO mice (Figure 3A), and total tau (Tau5) did not change with PTZ treatment in WT mice (Figure 3E). We used phosphorylated tyrosine residue of Src kinase [pSFK (pY416 in Fyn)] to determine the activation of Fyn relative to β-actin (Figures 3A,D). The pSFK levels were significantly upregulated in WT mice post-PTZ relative to the control (Figure 3D). In tau KO mice treated with PTZ, there was a significant reduction of pSFK levels compared to WT mice with PTZ. Although both Fyn KO and DKO mice still revealed pSFK expression to a degree, their levels were significantly lower relative to WT+PTZ mice (Figure 3D); however, they were not significantly different from WT control and tau KO mice (Figure 3D). Surprisingly, we did not observe an increase of total Fyn in the hippocampal lysates of WT mice treated with PTZ. Likewise, there was no difference in Fyn levels in tau KO mice between treatment groups (Figure 3F). Both Fyn KO and DKO mice did not show any traces of Fyn protein (Figure 3A). These results demonstrate that depleting Fyn or tau is sufficient to reduce hyperphosphorylated tau and Fyn activation in PTZ-induced seizures suggesting the plausible role of Fyn-tau interaction in the process of post-seizure epileptogenesis.

### Impact of PTZ-Induced Seizures on Microgliosis in KO and WT Mice

**Figure 4:**
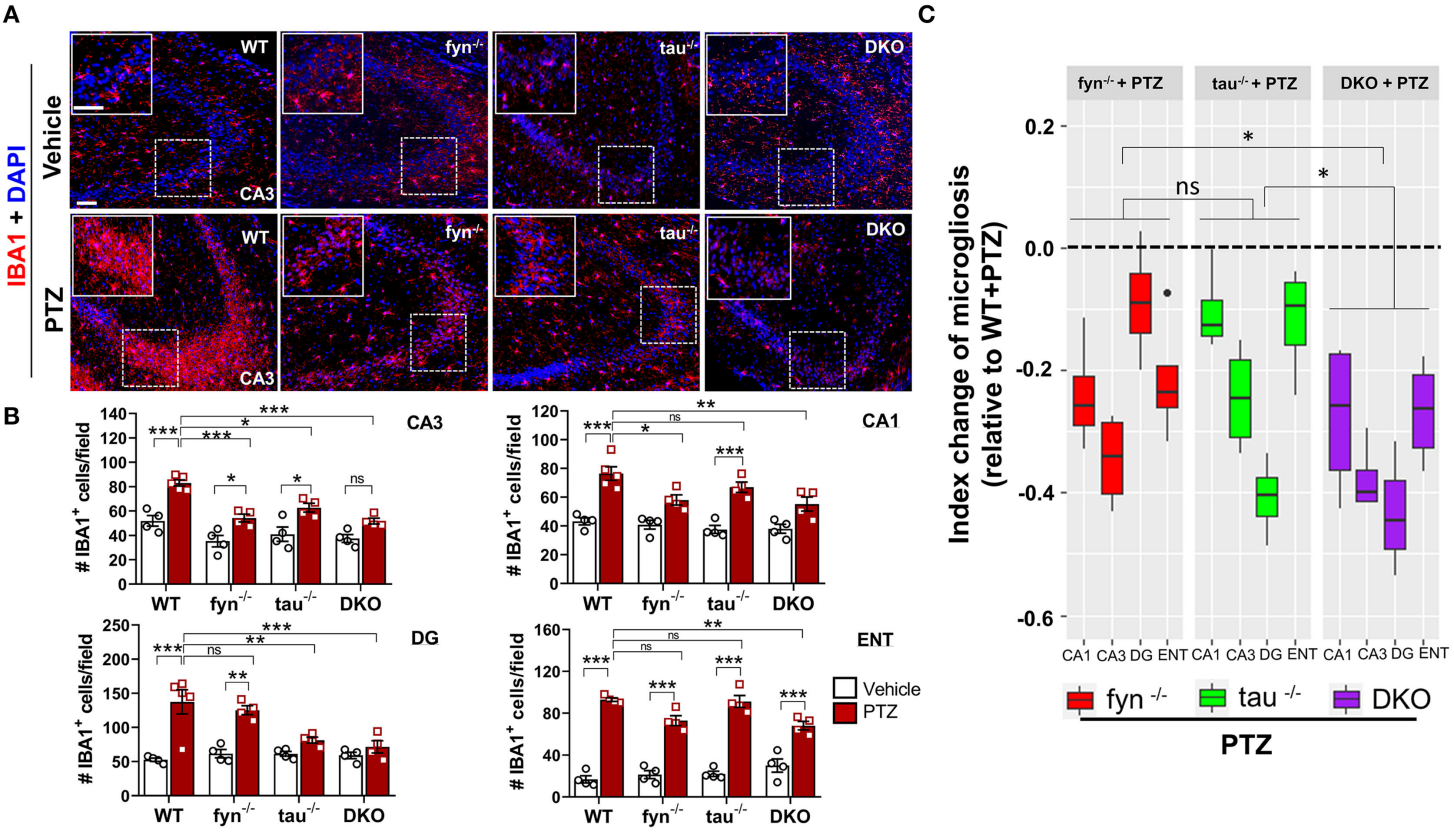
Absence of Fyn or tau or both reduced PTZ-induced microgliosis at 24 h post-seizure. **(A)** WT, *fyn*^−/−^, *tau*^−/−^, and DKO mouse brain sections were labeled with IBA1 (red) for microglia and counterstained with DAPI (blue). The large inset square at the upper left corner of each panel represents the enlarged area of the small square in dotted lines (representative images were shown). Scale bar, 100 μm. **(B)** Microglial cell quantification from CA1, CA3, DG, and ENT. The number of microglia was significantly increased in WT group as compared with its control in all regions. Both DKO and *fyn*^−/−^ demonstrated a significant reduction of microgliosis in CA3 and CA1 relative to WT. There was also a significant reduction in microgliosis in CA3 and DG in *tau*^−/−^ and DKO but not in *fyn*^−/−^. Only in DKO, microgliosis was significantly reduced in all four areas. **(C)** Linear mixed-effects analysis to measure index change of microglia count from *fyn*^−/−^, *tau*^−/−^, and DKO mice relative to WT. DKO showed more reduction in microgliosis relative to WT as compared to *fyn*^−/−^ and *tau*^−/−^ (*n* = 4–5, 4 sections/animal; ***p < 0.001, **p < 0.01, *p < 0.05, ns, not significant; two-way ANOVA with Tukey's *post-hoc* test for multiple comparisons). Data presented as mean ± SEM.

One of the prominent hallmarks of epileptogenesis is microgliosis and astrogliosis (Puttachary et al., 2016a; Sharma et al., 2018). We thus sought to examine the activated microglia in DKO and Fyn or tau KO mice following PTZ-induced seizures at 24 h. In the vehicle treated controls of all genotypes, IBA1 expressing microglia spread across the CA3 of the hippocampus with small cell body, long and branching processes. Following PTZ at 24 h, WT mice displayed a significant increase in the number of microglia in all three major regions of the hippocampus (CA1, CA3, and DG) and ENT (Figures 4A,B). A significant increase of microglia in CA3 was observed in Fyn KO and tau KO mice following PTZ administration (Figure 4B). In CA1, tau KO mice + PTZ also had significantly more IBA1^+^ cells, while Fyn KO mice + PTZ showed a significant decrease of microgliosis compared to WT+PTZ (Figure 4B). In DG, Fyn KO mice + PTZ had significant elevation of IBA1^+^ cells compared to controls while tau KO mice were not different from their controls but significantly reduced compared to WT+PTZ. In ENT, PTZ administration caused a significant increase of IBA1^+^ cells in all groups compared to their respective controls. On the other hand, although there was still a significant increase of microgliosis in ENT in DKO+PTZ mice, DKO mice were protected from PTZ-induced microgliosis in all regions of the hippocampus (Figure 4B). Moreover, only DKO mice group showed significantly reduced microglia compared to WT with PTZ in all four areas. An index change of microglia analysis revealed that DKO mice had more significant effects on the reduction of microgliosis than Fyn KO and tau KO mice while there was no difference between Fyn KO and tau KO mice (Figure 4C). Collectively, DKO mice had the most profound effects on the attenuation of microgliosis post-PTZ. Attenuation was seen in all regions compared to other KOs where suppression of microgliosis was region dependent.

### Fyn and Tau KO Prevented PTZ-Induced Microglial Polarization

**Figure 5:**
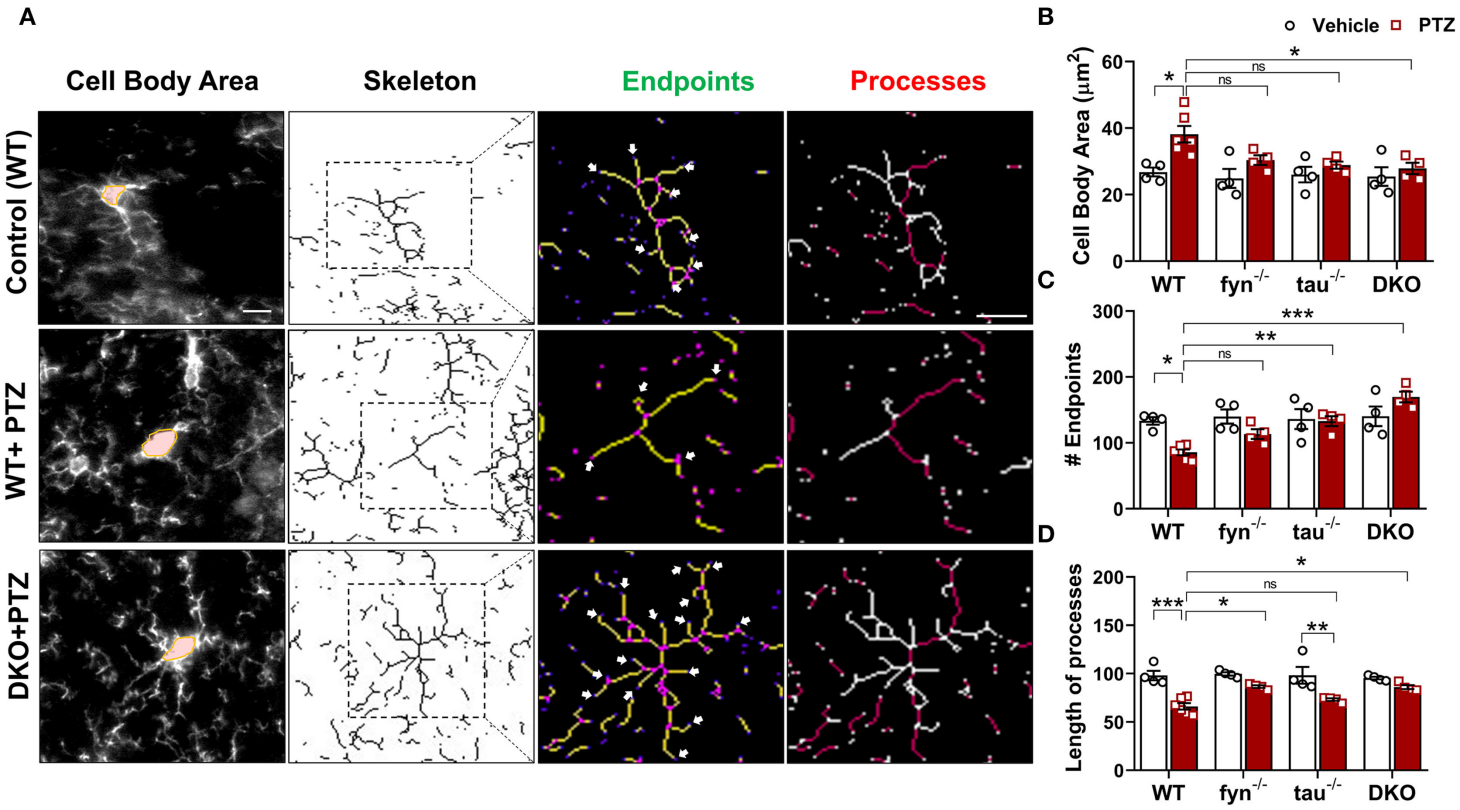
Fyn and tau loss prevented the polarization of microglia after PTZ-induced seizures. **(A)** A representative microglial skeletal analysis showing the images of IBA1 positive cells was captured with 40x objective. The cell body and the processes of microglia were measured. The number of endpoints (indicated by the arrows) and the two longest process paths (red processes) per microglia were quantified from >50 cells/group for statistical comparison across the groups. Scale bar 10 μm. **(B)** Cell body area was significantly increased in the WT and significantly reduced in DKO mice. **(C)** A significantly reduced number of microglial endpoints was found in the WT. Both *tau*^−/−^ and DKO, but not *fyn*^−/−^, showed significantly more endpoints than WT. **(D)** The length of the longest microglial processes was significantly reduced in WT and *tau*^−/−^, but they were unaffected in *fyn*^−/−^ and DKO (*n* = 4–6, 4 sections/animal; ***p < 0.001, **p < 0.01, *p < 0.05, ns, not significant; two-way ANOVA with Tukey's *post-hoc* test for multiple comparisons). Data presented as mean ± SEM.

Microglia have active and dynamic morphological transformations in response to alterations in the brain physiological state. The shapes of microglia can range from hyper-ramified to ameboid-like depending upon the severity of brain insult (Davalos et al., 2005). Since we observed a significant suppression of microgliosis in DKO, we extended our analysis to investigate subtle changes of microglial morphology in DKO compared to other KOs using the ImageJ skeleton analysis (Harvey et al., 2015; Morrison et al., 2017). Representative images of microglia with distinct morphological features in WT and DKO were captured and skeletonized (Figure 5A). The area of the cell body, the number of endpoints, and the length of longest processes were measured. Vehicle-treated controls for all genotypes had relatively small cell bodies, numerous endpoints, and long processes (Figure 5A). The microglia in WT mice treated with PTZ showed significantly larger cell body, fewer endpoints, and shorter processes than their control counterparts suggesting that following PTZ injection, the hippocampus of WT mice was vastly populated with amoeboid-like microglia (Figures 5B–D). Although microglial cell bodies from Fyn KO and tau KO mice treated with PTZ did not differ significantly from their controls, they were also not different from PTZ treated WT, despite 21 and 25% reduction of size in Fyn KO and tau KO, respectively (Figure 5B). The number of endpoints was significantly increased in tau KO+PTZ mice compared to WT+PTZ mice, while there was no significant difference between Fyn KO+PTZ mice and WT+PTZ mice (Figure 5C). Moreover, similar to those in WT, the length of the microglial process in tau KO was also reduced significantly after PTZ at 24 h when compared to its control. In contrast, Fyn KO+PTZ mice had significantly longer processes than WT+PTZ mice (Figure 5D). On the other hand, PTZ-treated DKO mice had no difference in microglial morphology from vehicle-treated controls. In comparison with WT+PTZ mice, DKO mice had significantly smaller cell bodies, more endpoints, and longer processes indicating highly ramified microglia in DKO mice post-PTZ, whereas single KOs were only significantly different in some aspects (Figures 5A–D). These results suggest that disabling both Fyn and tau altogether prevents polarization of microglia despite exposure to a chemoconvulsant.

### Impact of PTZ-Induced Seizures on Astrogliosis in KO and WT Mice

**Figure 6:**
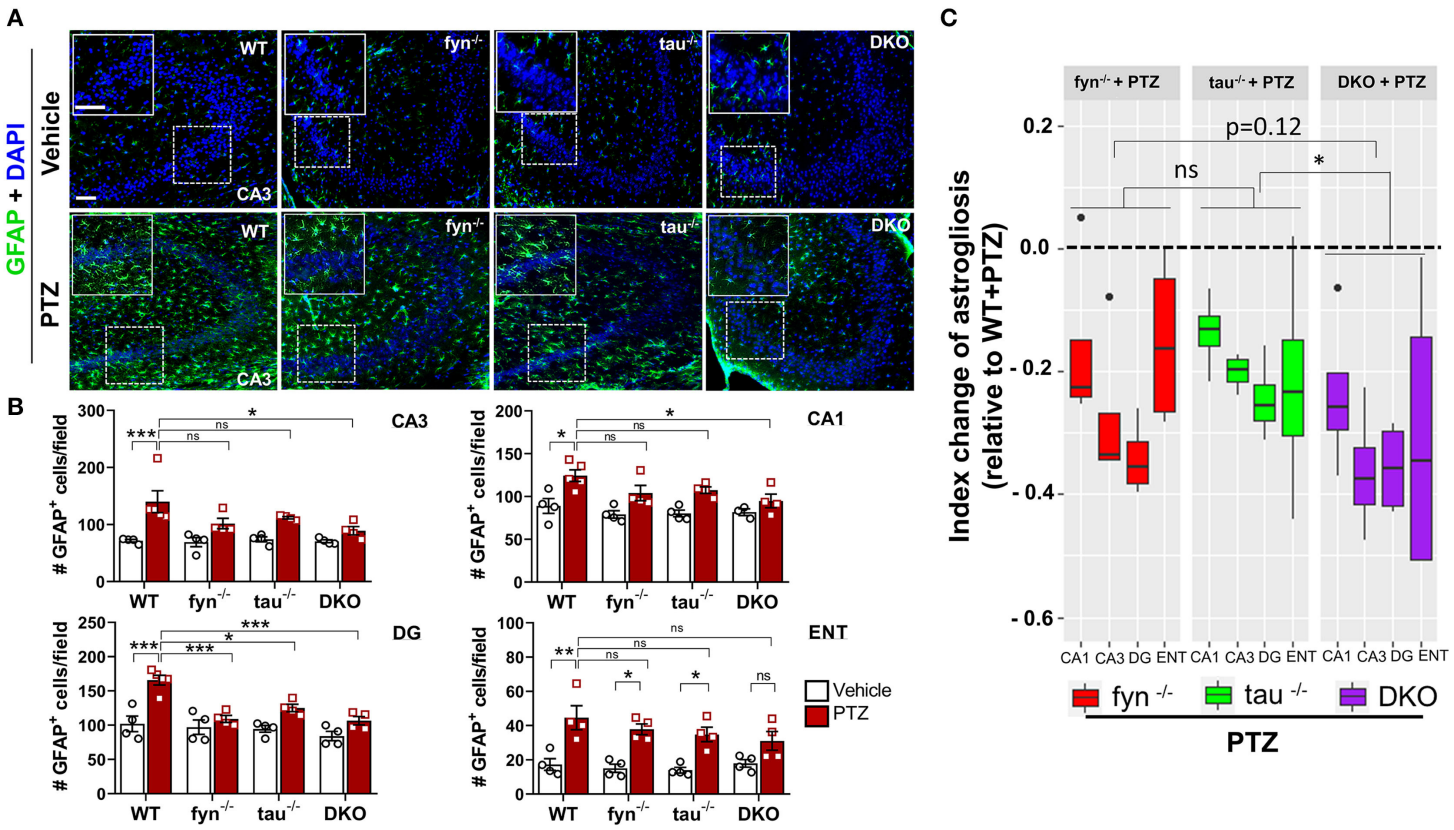
Absence of Fyn or tau or both reduced PTZ-induced astrogliosis at 24 h post-seizure. **(A)** WT, *fyn*^−/−^, *tau*^−/−^, and DKO mouse brain sections were labeled with GFAP (green) for astrocytes and counterstained with DAPI (blue). The large inset square at the upper left corner of each panel represents the enlarged area of the small square in dotted lines. **(B)** Astrocyte cell quantification. The numbers of GFAP positive cells were significantly increased in WT group in all regions of the hippocampus and ENT following PTZ injection. DKO revealed a significant reduction of astrogliosis in all regions except for ENT. Both *fyn*^−/−^ and *tau*^−/−^ had a significant reduction of astrocytes in DG only. **(C)** Linear mixed-effects analysis to measure index change of GFAP positive cell count from *fyn*^−/−^, *tau*^−/−^, and DKO mice relative to WT. DKO was significantly different from *tau*^−/−^, but not from *fyn*^−/−^ in terms of effects on reduction of astrogliosis (*n* = 4–5, 3–4 sections/animal; ***p < 0.001, **p < 0.01, *p < 0.05, ns, not significant; two-way ANOVA with ANOVA with Tukey's *post-hoc* test for multiple comparisons). Data presented as mean ± SEM.

Intense seizure activities are highly correlated with increased astrogliosis, which mediates neuronal hyperexcitability in patients with epilepsy (Devinsky et al., 2013). To investigate the distribution patterns of astrogliosis following SE in PTZ-induced seizures, we performed IHC with astroglial marker, GFAP (Figure 6A). We found that, at 24 h PTZ-induced seizures, in the hippocampus and ENT, WT mice showed a robust increase of GFAP staining intensity with distinct morphological features such as a larger cell body and more hypertrophic processes compared to the vehicle treated control WT mice (Figure 6B). Fyn KO and tau KO mice showed a significant reduction of GFAP intensity in DG following PTZ injections compared to their controls. There was no significant difference between WT and either Fyn KO or tau KO in CA3 and CA1 of the hippocampus and ENT (Figure 6B). Moreover, both Fyn KO and tau KO mice had a significant increase of GFAP positive cells compared to their controls in ENT (Figure 6B). DKO mice significantly prevented astrogliosis in all regions of the hippocampus. Despite having no statistically significant reduction of GFAP+ cells in comparison to WT+PTZ mice in ENT region, DKO mice with PTZ were not different from vehicle treated DKO mice suggesting that DKO prevented reactive astrogliosis to some extent (Figure 6B). Thus, to measure the overall protective effects of all KOs following PTZ injection, index change of astrogliosis relative to WT+PTZ mice was plotted for each group. A mixed-effects model analysis showed that DKO mice had significantly more effects on the reduction of astrogliosis than in tau KO mice (*p* = 0.0496). Compared to Fyn KO mice, no difference was observed despite the trend of more decrease of value in DKO mice than in Fyn KO mice (*p* = 0.125) (Figure 6C). Overall, these data indicate that disabling Fyn and tau prevents astrogliosis in PTZ-induced seizures.

### Fyn and Tau KO Prevented the Loss of Astrocytic Kir 4.1 Levels in PTZ-Induced Seizures

**Figure 7:**
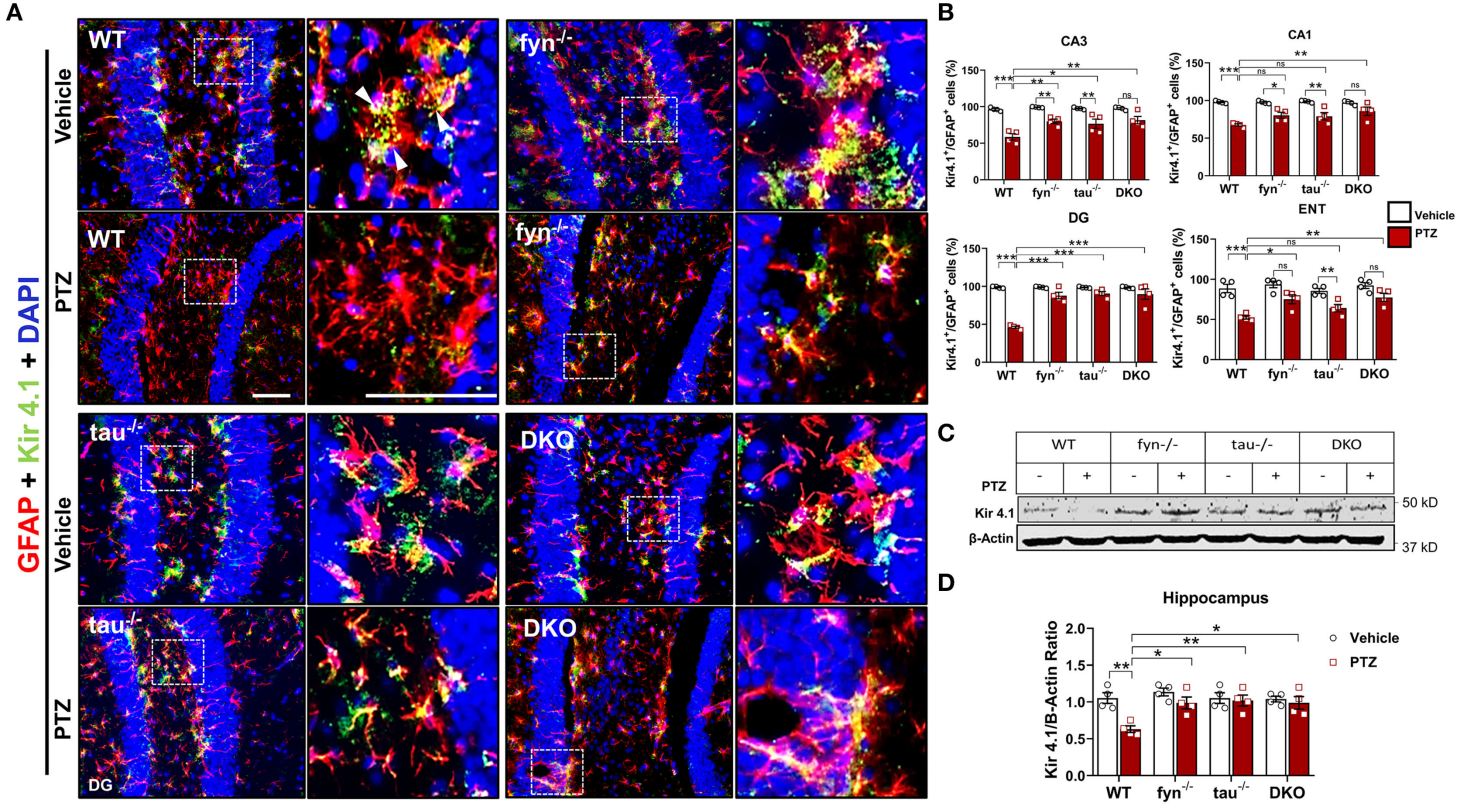
Expression of Kir 4.1 was protected in Fyn and tau KO mice at 24 h after seizure induction. **(A)** Brain sections were double labeled for Kir4.1 and GFAP (Representative images of DG were shown). The magnified images in the second and fourth columns represent the squares in each adjacent panel. Scale bar, 100 μm. **(B)** Cell quantification shows the percentage of Kir 4.1 and GFAP co-localization in the hippocampus and ENT. DKO mice demonstrated significant protection against Kir 4.1 loss post- PTZ as compared to WT in all regions. Regionally, *fyn*^−/−^ and *tau*^−/−^ mice had a significant increase of Kir 4.1 expression in CA3 and DG. In addition, *fyn*^−/−^ mice also showed a significant rescue of Kir 4.1 in ENT. **(C)** A representative Western blot of hippocampal lysates showing reduced expression of Kir 4.1 in WT following PTZ injection. In contrast, its levels were retained in all KO mice. **(D)** Quantified WB results of all groups are shown (*n* = 4; ***p < 0.001, **p < 0.01, *p < 0.05; ns, not significant; two-way ANOVA with Tukey's *post-hoc* test for multiple comparisons). Data presented as mean ± SEM.

One of the major functions of astrocytes is to regulate K^+^ homeostasis at the synaptic cleft through inwardly rectifying K^+^ channels (Kir 4.1). Several studies have reported markedly reduced expression of Kir 4.1 in animal models of epilepsy and human epilepsy (Heuser et al., 2012; Puttachary et al., 2016b). Since we found a significant decrease in astrogliosis in DKO mice following PTZ-induced seizures, we then further investigated Kir 4.1 levels in astrocytes. Representative IHC images from all genotypes showed robust co-localization of Kir 4.1 and GFAP^+^ cells in the DG of hippocampus except in WT mice with PTZ that appeared to have less Kir 4.1 expression, but more GFAP+ cells (Figure 7A). Quantitatively, both PTZ treated Fyn KO, and tau KO mice had a significant downregulation of Kir 4.1 compared to their controls in CA3 (Figure 7B). In DG and CA3, both Fyn KO and tau KO mice also showed significantly more Kir 4.1 levels than in PTZ-treated WT mice. In ENT, Fyn KO mice had a significant increase of Kir 4.1 levels compared to WT+PTZ mice, while tau KO+PTZ mice were not different from WT+PTZ mice and had significantly lower expression than vehicle-treated tau KO (Figure 7B). As shown in representative Western blot (Figure 7C), we found a significant reduction in Kir 4.1 in WT with PTZ samples while its levels were preserved in all KOs group treated with PTZ (Figure 7D). In DKO+PTZ group, they had significantly higher Kir 4.1 expression in CA1, CA3, DG, and ENT than WT+PTZ mice, indicating that DKO retains the normal levels of Kir 4.1 in all four areas, although they were statistically different from other single KOs. Overall, our data suggest that reducing Fyn and tau prevents the loss of Kir 4.1 channels in astrocytes in the acute phase of epileptogenesis following PTZ-induced seizures.

### Fyn and Tau KO Protected Against PTZ-Induced Neurodegeneration and Loss of Parvalbumin Interneurons

**Figure 8:**
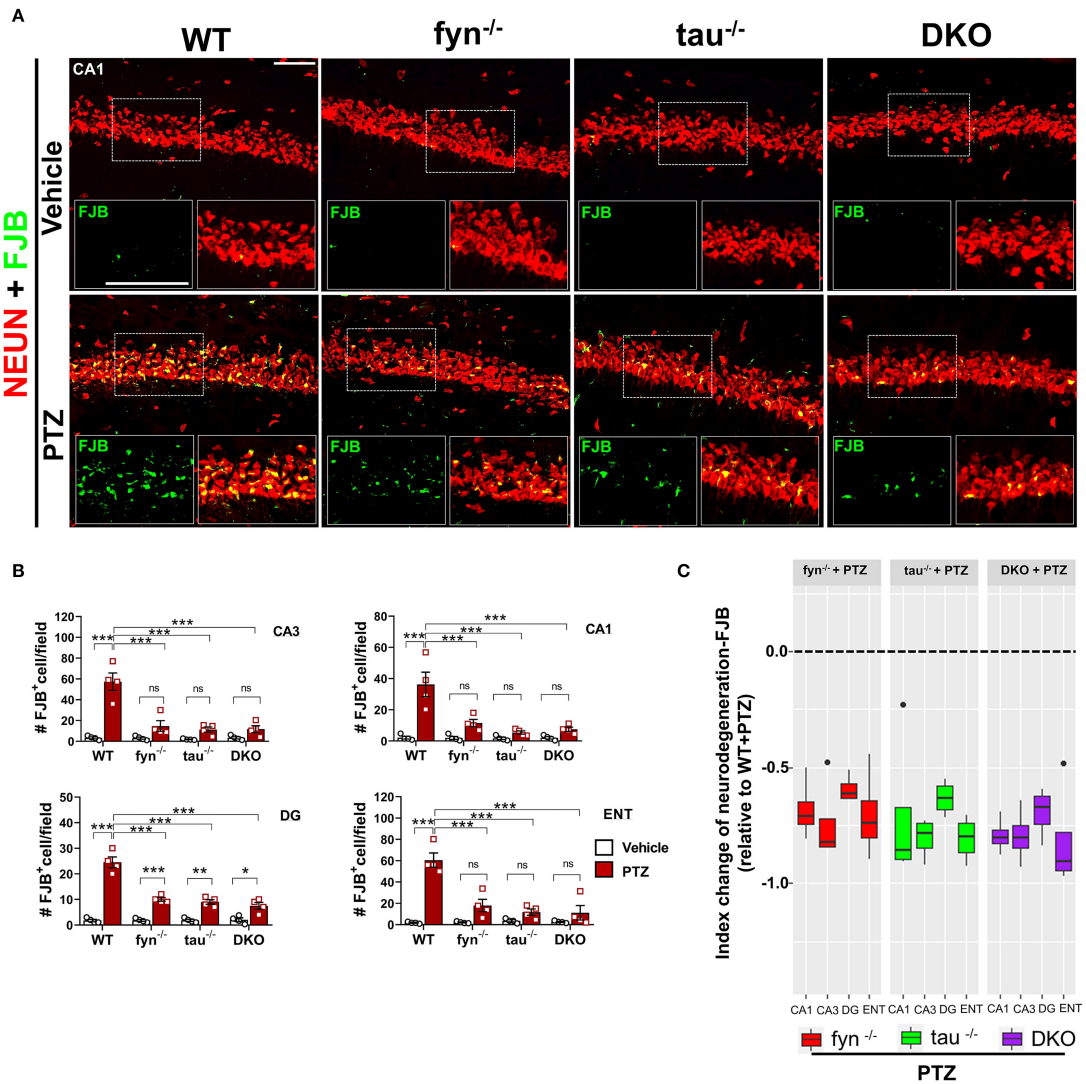
Reduced neurodegeneration in Fyn and tau KO mice in PTZ-induced seizures. **(A)** WT, *fyn*^−/−^, *tau*^−/−^, and DKO mouse brain sections were labeled for Fluoro-Jade B (FJB) and NeuN, showing extensive neurodegeneration in CA1 of WT in contrast to KO mice following PTZ. The large inset square in the lower left (FJB only- green labeled cells) and right (merged NeuN+FJB- yellow labeled neurons) corner of each panel represents the enlarged area of the small square in dotted lines. Scale bar 100 μm. **(B)** Quantification of FJB positive cells in the hippocampus and ENT. Although all KOs showed a degree of neurodegeneration following PTZ injection as compared to their respective controls, the significant difference was only found in DG. Neurodegeneration was significantly reduced in all KO mice compared with WT following PTZ. **(C)** Linear mixed-effects analysis to measure index change of FJB+NeuN count from *fyn*^−/−^, *tau*^−/−^, and DKO mice relative to WT. There was no difference between KOs in FJB positive neurons (*n* = 4, 3–4 sections/animal; *p < 0.05; **p < 0.01; ***p < 0.001; ns, not significant; two-way ANOVA with Tukey's *post-hoc* test for multiple comparisons). Data presented as mean ± SEM.

Neurodegeneration is one of the most common consequences of seizures that could lead to aberrant neuronal network formation resulting in more seizures generation (Lado et al., 2000; Naegele, 2007). We then investigated whether DKO is capable of rescuing neurodegeneration and loss of GABAergic interneurons following PTZ-induced seizures. Co-labeling of a neuronal marker, NeuN, and Fluoro-Jade B (FJB), a marker for dying neurons (Figure 8A), demonstrated a significant increase of number of NeuN+FJB cells in hippocampus and ENT in PTZ treated WT mice compared to WT control (Figure 8B). This suggests that PTZ-induced seizures caused massive neurodegeneration at 24 h post-treatment. Although all KOs still showed an increase of dying neurons to an extent, particularly significant in DG (Figure 8B), as compared to their controls in response to PTZ-induced seizures, they showed significantly less FJB^+^ cells in all regions compared to WT+PTZ mice, suggesting neuroprotective roles of disabled Fyn-tau in mitigating seizure-induced neurodegeneration (Figure 8B). To measure the overall neuroprotective effects against neurodegeneration in all KOs, an index change of FJB cell count relative to WT was plotted for Fyn KO, tau KO, and DKO mice. An index change of neurodegeneration analyzed with a linear mixed-effect model revealed no difference between DKO, Fyn KO, and tau KO mice treated with PTZ regarding the overall neuroprotective effects against PTZ-induced neurodegeneration (Figure 8C).

**Figure 9:**
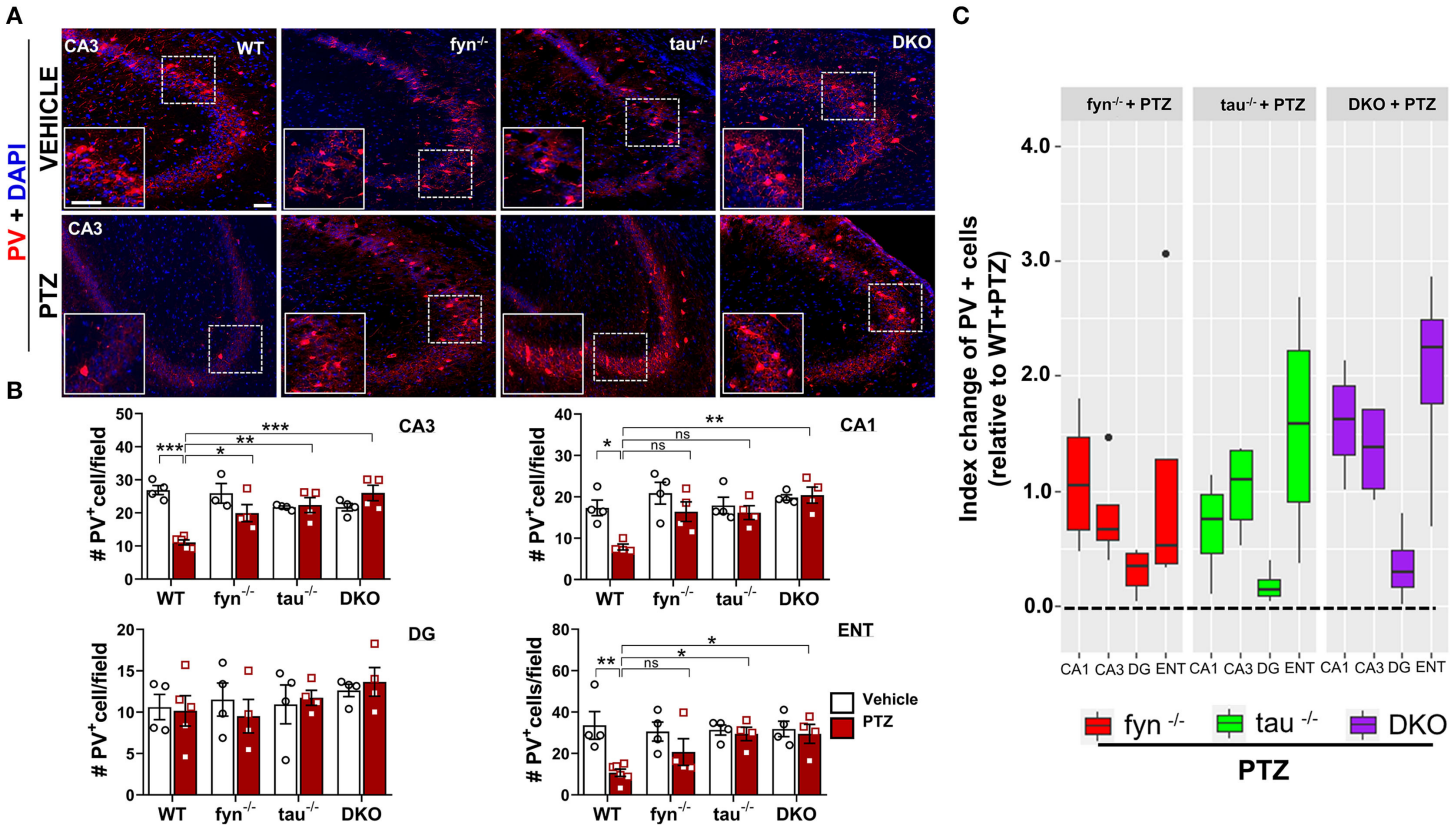
PTZ-induced loss of parvalbumin (PV) expressing interneurons was protected in Fyn and tau KO mice 24 h after seizures. **(A)** PV immunostaining of brain sections from all groups in CA3 of the hippocampus. The large inset square in the lower left in each panel represents the enlarged area of the small square in dotted lines (representative images are shown). Scale bar, 100 μm. **(B)** Quantification of PV-expressing cells in the hippocampus and ENT showed reduced PV cells in WT while in DKO, its levels were not reduced when compared with their untreated PTZ controls. Both *fyn*^−/−^ and *tau*^−/−^ showed a significant increase of PV in CA3 while tau KO, but not *fyn*^−/−^ also significantly rescued the loss of PV in ENT compared to WT. **(C)** Linear mixed-effects analysis to measure index change of PV positive cell count from *fyn*^−/−^, *tau*^−/−^, and DKO mice relative to WT. There was no difference between KOs in terms of effects on protecting PV interneurons (*n* = 4–6, 3–4 sections/animal; ***p < 0.001, **p < 0.01, *p < 0.05, ns, not significant; two-way ANOVAs were used for statistical analysis followed by Tukey's multiple comparisons test). Data presented as mean ± SEM.

We further evaluated the effects of disabling Fyn and tau on the expression of GABAergic interneurons in response to PTZ-induced seizures using a specific marker, calcium-binding protein parvalbumin (PV) (Figure 9A). A significant loss of PV interneurons was observed in WT mice at 24 h post-PTZ administration when compared with vehicle-treated WT mice (Figures 9A,B). Differences between Fyn KO mice and WT mice treated with PTZ were only observed in CA3, while tau KO mice treated with PTZ had significantly more PV^+^ interneurons in CA3 and ENT relative to WT+PTZ group (Figure 9B). DKO mice treated with PTZ had significantly more PV^+^ interneurons in CA3, CA1, and ENT as compared to WT treated with PTZ. Interestingly, in DG, PTZ did not affect the number of PV^+^ interneurons across all four genotypes. Moreover, a mixed-effect model showed no overall differences on index change of PV cells between Fyn KO, tau KO, DKO groups, despite there being a trend increase of value overall in DKO mice relative to other KOs [DKO vs. Fyn KO (*p* = 0.0925)], DKO vs. tau KO (*p* = 0.1219) (Figure 9C). Collectively, these data suggest that Fyn and tau play a major role in facilitating neurodegeneration following PTZ-induced seizures, thus combinatorially ablating Fyn and tau is neuroprotective.

## Discussion

In this study, we provide evidence for the role of Fyn-tau interaction and the impact of the absence of Fyn or tau or both in seizure susceptibility and subsequent neurobiological changes in the brain using gene KO mouse injected with PTZ. In neurological diseases that manifest seizures, tau loss attenuates resistance to seizure induction (Ittner et al., 2010; DeVos et al., 2013) while Fyn reduction not only reduces seizures and seizure-induced mortality (Sharma et al., 2018), but also suppresses tau pathology, neuroinflammation, and cognitive deficits (Kaufman et al., 2015; Tang et al., 2020). Complete tau ablation in genetic models of epilepsy significantly improved the disease outcome (Holth et al., 2013; Gheyara et al., 2014). Evidence underlining the role of tau aggregates in seizures has emerged from independent studies of human epileptic brains (Tai et al., 2016; Gourmaud et al., 2020) and experimental model of TLE (Alves et al., 2019). Although the role of Fyn in seizures has not been reported in human epilepsy, an animal model of temporal lobe of epilepsy (TLE) showed that high expression of Fyn in the hippocampus (Sharma et al., 2018) or genetically manipulated active form of Fyn (Kojima et al., 1998) are significantly associated with high seizure incidents. Increased Fyn expression and its co-localization with tau in the hippocampus have been reported from human AD patients (Shirazi and Wood, 1993). Spontaneous seizure occurrence and hippocampal degeneration are the most common clinical sign and brain pathology in severe AD patients, raising the possibility that Fyn and tau might be involved in facilitating these abnormalities. Therefore, characterizing the effects of combinatorial Fyn and tau ablation in a seizure model and its impact on the early markers of epileptogenesis will provide insight for early intervention strategy. Our study demonstrates the protective effects in the absence of Fyn and tau in response to PTZ-induced seizures and brain pathology.

DKO mice showed significantly increased latency to convulsive seizures and overall reduced seizure severity, but they are not so different from tau KO mice (Figure 1). Another study also corroborated the protective effects of combinatorial Fyn and tau removal against PTZ induced-seizures (Liu et al., 2019). While tau KO mice were similar to DKO mice regarding protective effects against seizures, the Fyn KO mice did not significantly display sufficient anti-seizure effects despite having a reduction of seizure severity by 36 ± 8% compared to WT mice treated with PTZ in this study (Figures 1C–E). Fyn KO mice also showed no significant differences in seizure latency as compared to WT mice. Though these findings seem to contradict our previous Fyn KO studies (Sharma et al., 2018), a majority of the Fyn KO mice that died due to seizures in this study had severe hydrocephalus. The Fyn KO mice used in Sharma et al. were bred on Balb-c x C57BL/6J background in contrast to S129 x C57BL/6J background mice used in this study. There was no study, however, reported examining the mouse genetic background Balb-c vs. S129 in relation to the incidence of hydrocephalus. Furthermore, a repeated-low-dose of KA method, which induces prolonged seizures, was used by Sharma et al. in contrast to the PTZ method, which only induces transient seizures. Having known that hydrocephalous was a problem in Fyn KO mice in this study, hydrocephalous-free mice identified by MRI were subsequently used for the production of DKO. A previous study using the same colony of mice, but with larger sample size and hydrocephalous-free mice, also conferred a milder anti-seizure effect of Fyn KO relative to the tau KO and DKO mice (Liu et al., 2019). The reduced seizure severity in response to PTZ was similar in tau KO and DKO, which may imply that the protective effects could be predominantly due to the effects of tau KO in DKO, considering the marginal effects of Fyn KO in these strains. This also indicated that anti-seizure mechanisms in tau KO might be independent of Fyn and suggested a similar anti-seizure mechanism in DKO mice that was primarily driven by tau depletion. Earlier studies also found that Fyn reduction did not affect seizure threshold (Kojima et al., 1998) while others observed higher seizure susceptibility in Fyn-deficient mice (C57BL/6 × CBA F1) (Miyakawa et al., 1995, Miyakawa et al., 1996), which could be due to brain malformation rather than the direct effects of the absence of Fyn. We did not observe any spontaneous seizures in hydrocephalic Fyn KO mice during handling or during routine husbandry. However, we did not conduct long-term video-EEG studies in these hydrocephalic Fyn KO mice, without PTZ, to confirm whether they were spontaneously seizing or not. Liu et al. reported that hydrocephalic Fyn KO mice had significantly higher seizure susceptibility than WT mice with PTZ treatment while non-hydrocephalic Fyn KO mice were protected against seizures (Liu et al., 2019). Therefore, the difference in mouse genetic background and the incidence of hydrocephalous could be an underlying cause for variable responses of Fyn KO to seizure in this study. Despite conflicting results from Fyn KO mice, pretreatment of wild type C57BL/6J mice with Src kinase inhibitor, Saracatinib, showed a significant reduction in seizure severity when compared with vehicle-treated mice in a KA model (Sharma et al., 2018), suggesting the role of Fyn and other Src kinases in seizures. Apart from Fyn inhibition/Fyn KO or tau KO or DKO, neuroprotective effects can also be achieved through targeting other pathways such as Ras inhibitor (Bi et al., 2017) or K^+^ voltage-ion channels, Kv4.2 (Hall et al., 2015) that are independent of Fyn. In addition to seizure phenotypes, both Fyn KO and DKO mice had cognitive deficits in the novel object test. In the contextual fear conditioning test, Fyn KO and DKO exhibited learning deficits, although there was no differences in memory among the genotypes relative to the WT mice. Aged tau KO and DKO mice showed reduced motor function compared to age-matched WT mice in the pole test. Behaviorally, it appears that tau is critically involved in motor function while Fyn is strongly attributed to cognitive tasks (Liu et al., 2019).

At the synaptic level, the phosphorylated tau serves as a chaperone to localize Fyn in the post-synaptic terminal (Ittner et al., 2010; Ittner and Götz, 2011). Activated Fyn also phosphorylates tau at tyrosine residue, Y18, which is also present in concomitant with abnormally phosphorylated tau in tau-related disorders (Lee et al., 2004; Bhaskar et al., 2005). Upon localization of Fyn-tau complexes in dendritic spines, Fyn then phosphorylates the NR2B subunit of NMDAR at Tyr 1472, facilitating the interaction of PSD-95 and NR2B to stabilize the NR2B/PSD-95/tau/Fyn complex (Ittner and Götz, 2011). Activated NMDAR overloads Ca^2+^ and Na^+^ into the neurons leading to neuronal hyperexcitability and neurodegeneration (Ittner et al., 2010). In KA-induced seizure model, a 3-4 fold increase in tau hyperphosphorylation at the Ser199/Ser 202 epitopes (AT8) was observed within a few hours after acute seizures and persisted in the epileptic phase (Liang et al., 2009; Alves et al., 2019). An overexpression of neuronal Fyn increases tau hyperphosphorylation on AT8 (Xia and Götz, 2014) while Fyn-tau interaction increases with tau phosphorylation that would then cause neurodegeneration (Bhaskar et al., 2005; Mondragón-Rodríguez et al., 2012). In concurrence with these reports, we also observed increased co-expression of AT8 and Fyn in the hippocampus and ENT at 24 h in PTZ- induced seizures (Figure 2). Likewise, our Western blot (WB) analysis of the whole hippocampal lysates showed not only increased levels of AT8 but also upregulation of pY18, a phospho-tau by Fyn, and activated Fyn (pSFK) in WT mice treated with PTZ at 24 h (Figure 3). Surprisingly, the hippocampal WB did not show an increase of native Fyn protein in WT mice post-PTZ, indicating that increased Fyn might occur in a region-specific manner as shown by IHC (Figure 2B). Furthermore, it could be due to the limited duration of initial seizures for <10 min in PTZ model, in contrast, to >40 min in KA model (Sharma et al., 2018). We also found a significant increase of Fyn-tau interaction in the hippocampus, detected by PLA, following PTZ-induced seizures (Figures 2D,E). All these results suggest the role of the pathogenic form of Fyn and tau in seizure generation. Therefore, we speculated that Fyn-tau complexes could mediate seizure-induced neurotoxicity observed in our model. Furthermore, single Fyn KO and tau KO downregulated the increased levels of AT8, pY18, and pSFK following PTZ injection (Figure 3), which is also in line with a recent study showing a reduction of tau phosphorylation with Fyn depletion (Liu et al., 2020). It is clear that knocking out either Fyn or tau by means to disrupt the interaction is sufficient to suppress tau pathology and Fyn activation. Therefore, strategies that aim to disrupt Fyn-tau interaction could be a novel potential therapeutic approach to modify or prevent disease progression (Rush et al., 2019). One therapeutic approach would be by either inhibiting Fyn (Sharma et al., 2018) or reducing tau (DeVos et al., 2013; Tang et al., 2020). A recent study showed that a peptide that prevented the interaction of Fyn with tau rescued neurodegeneration and associated neuronal toxicity in an *in vitro* Aβ-amyloid primary neuronal culture (Rush et al., 2019). In addition, a recent study demonstrated that pharmacological inhibition of Fyn decreased Fyn and tau interaction in a mouse model of tauopathy and prevented neurodegeneration resulting in improved learning and memory (Tang et al., 2020). Other approaches, such as inducing site-specific tau phosphorylation at Thr205 or blocking PSD-95 using tat-NR2B9c peptide have been reported to dissociate NR2B/PSD-95/tau/Fyn complexes leading to the rescue of neurotoxicity (Ittner et al., 2016; Tse et al., 2019).

There is still a paucity of information, however, on the roles of Fyn and tau in seizure-mediated neuroinflammation. Hyperphosphorylated tau can induce microglial and astrocyte activation leading to persistent neuroinflammation in various tau-associated disorders and aid in the spread of tau pathology (Bhaskar et al., 2010; Maphis et al., 2015b; Laurent et al., 2018). In addition, the loss of tau has been reported to alleviate microgliosis (Maphis et al., 2015a). A previous study showed an increase of Fyn activation in KA-induced TLE model (Sharma et al., 2018). While overexpressing Fyn, not only increases tau phosphorylation (Xia and Götz, 2014), but also exacerbates inflammatory state of the brain (Panicker et al., 2019), reducing Fyn, either genetically or pharmacologically, attenuated microgliosis (Panicker et al., 2015, 2019; Sharma et al., 2018). In regards to astrocyte, Fyn inhibition ameliorated the hyperphosphorylated tau-induced astrogliosis (Tang et al., 2020), whereas reducing tau is protective against reactive astrocytes (DeVos et al., 2018). Gliosis has been considered the hallmark of brain inflammation, which contributes to the production of proinflammatory cytokines and neurodegeneration in epilepsy and seizure models (Devinsky et al., 2013; Vezzani, 2014; Sharma et al., 2018; van Vliet et al., 2018; Vezzani et al., 2019). We and others have demonstrated that the activation of glial cells, in both *in vivo* and *in vitro*, increases the production of pro-inflammatory cytokines and chemokines which are detrimental to neurons (Arisi et al., 2015; Panicker et al., 2015; Cerri et al., 2016; Tian et al., 2017; Rana and Musto, 2018; Putra et al., 2020b). Although we did not directly measure the pro-inflammatory cytokines in this study, reactive gliosis and neurodegeneration were observed in the WT mice challenged with PTZ, while the Fyn, tau, and DKO mice were protected (Figures 4, 6, 8). Relative to DKO, Fyn KO, and tau KO mice only showed milder protection against the neuroinflammatory milieu in some regions (Figures 4, 6). Meanwhile, combinatorial Fyn and tau ablation with DKO mice protected all regions from microgliosis and astrogliosis following PTZ, indicating the superiority of DKO mice over single KOs in mitigating seizure-induced progressive neuroinflammation. DKO mice significantly reduced microgliosis compared to both single KOs (Figure 4C), suggesting a synergistic role of Fyn and tau in facilitating microgliosis. Overall, these data demonstrate the effects of both Fyn and tau reduction in accomplishing protection against PTZ-induced neuroinflammation.

Brain inflammation, in response to an insult such as seizures, impacts microglial behavior to initially supporting the brain, depending on the degree of insult (Davalos et al., 2005). Other studies have reported that microglia can transform into multiple shapes ranging from complex, hyper-ramified to ameboid-like cells (Karperien et al., 2013). Currently, little is known about the dynamics of the morphological changes in microglia following seizures, and reliable markers for reactive microglia are still not available. While we and others have previously used CD68 as a marker for reactive microglia (Zhao et al., 2018; Putra et al., 2020b), CD68 is also expressed in other types of microglia. In this study, therefore, we did a skeletonized morphometric analysis to determine their active state (Morrison et al., 2017). In this analysis, we found that reactive microglia were characterized by the large cell bodies, fewer branches, and short processes that matched with amoeboid-shaped cells in WT mice following PTZ (Figure 5). We found that only in DKO mice, there was a complete restoration of microglial morphology following seizures. In DKO, despite treating with PTZ, microglia maintained the surveilling and ramified state with small cell body (Figure 5B), more branches (Figure 5C), and long and intricate processes (Figure 5D). Unlike in DKO mice, single KO mice Fyn and Tau KO only rescued the microglial morphology to a lesser extent, indicating that single KOs were inadequate to completely prevent the polarization of microglia after PTZ (Figures 5B–D). While pro-inflammatory cytokine profiling was not done in this study, the morphometric analysis suggested that eliminating both Fyn and tau suppresses microgliosis and prevents polarization. Nevertheless, the underlying molecular mechanisms of these processes remain elusive and warrants further investigation.

In contrast to microglia, seizure-induced changes in astrocyte numbers and morphology in different brain regions have been extensively studied (Wetherington et al., 2008; Martinian et al., 2009; Das et al., 2012; Puttachary et al., 2016a,b). In reactive astrocytes, we and others had demonstrated the downregulation of astrocytic Kir 4.1, inwardly rectifying K^+^ channels, in KA model of chronic epilepsy (Zurolo et al., 2012; Puttachary et al., 2016b). Reduction in Kir 4.1 results in the inability of astrocytes to regulate K^+^ at synaptic clefts and exacerbates hyperexcitability of neurons and neurodegeneration (Devinsky et al., 2013). Kir 4.1 has been found to be downregulated in both the acute seizures animal model (Zurolo et al., 2012) and human epileptic brain (Das et al., 2012; Heuser et al., 2012). In the absence of reliable markers for reactive astrocytes, we, therefore, performed Kir 4.1 IHC and WB analyses to understand the role of astroglial function in our model. Hitherto, it was unknown, however, whether Fyn and/or tau loss would alter the levels of Kir 4.1. Our data demonstrated that while PTZ-treated WT exhibited lower expression of Kir 4.1, loss of Fyn or tau or both did not affect Kir 4.1 expression post-PTZ (Figure 7), suggesting the maintenance of K^+^ buffering in KO mice. Although there were differences in the degree of significance in regard to Kir 4.1 levels between KO groups across the regions (Figure 7B), hippocampal WB showed relatively same levels of Kir 4.1 between DKO, Fyn KO, tau KO post-PTZ administration (Figure 7C). The mechanisms, however, by which Fyn and tau modulate the function of astrocytes and affect the expression levels of astrocytic Kir 4.1 remain poorly understood. In addition, Fyn activation and tau pathology have been reported to induce astrogliosis leading to the alteration of synaptic homeostasis and neuronal network dysfunctions in transgenic AD models (Kaufman et al., 2015). Thus, preventing seizure-induced neurotoxicity through depletion of Fyn and/or tau may indirectly inhibit K^+^ buffering dysfunction in astrocytes.

Neurodegeneration is one of the most common features of many epilepsies, contributing to the hyperexcitable neuronal network (Lado et al., 2000; Naegele, 2007). The role of Fyn-tau interaction in NMDAR-mediated neurodegeneration has been known for some time (Bhaskar et al., 2005; Ittner et al., 2010; Ittner and Götz, 2011). Consistent with these studies, we found an increase of neuronal death, measured by FJB staining, in the hippocampus and ENT of WT mice treated with PTZ. Compared to WT mice, Fyn KO, tau KO, and DKO mice were protected from neurodegeneration (Figures 8A,B), implicating that inhibiting Fyn/tau complexes in neurons thorough either individual ablation of Fyn or tau or combination can be neuroprotective. Moreover, a mixed-effect model measuring the overall neuroprotection effects in each KOs (Figure 7C) showed that DKO mice were not different from Fyn KO and tau KO, suggesting that DKO were not superior to Fyn or tau KO alone, thus reducing either of the two protein is sufficient to reduce neuronal death. Of note, there was a loss of PV-interneurons in the hippocampus, a subset of GABAergic interneurons, following PTZ-induced seizure in WT mice (Figure 9). A similar finding was reported in both rat and mouse seizure models (Martin and Sloviter, 2001; Marx et al., 2013). Surprisingly, PV interneurons in DG were unaffected by PTZ injection across all KO and WT groups (Figure 9B). While Fyn KO and tau KO mice were protected from loss of PV interneurons in some regions, DKO mice appeared to show the most neuroprotective effect on PV interneurons in the hippocampus and ENT relative to other single KOs (Figure 9B). However, when analyzed globally across all regions, DKO mice did not differ from Fyn KO or tau KO (Figure 9C), indicating the superior protection of DKO was limited to specific brain regions. It is speculative that seizures induce hyperphosphorylated tau that leads to the death of GABAergic interneurons. In support of this, a study with the AD mouse model that exhibited seizures showed a significant loss of PV interneurons (Levenga et al., 2013). Nevertheless, the mechanism of Fyn and tau mediated PV interneurons death in specific brain regions requires further investigation.

In conclusion, we demonstrated the common consequences of post-seizures such as neurodegeneration and gliosis in a PTZ mouse model, though the continuous seizures duration was <10 min. PTZ-induced acute seizure model also confirmed Fyn or tau hyperphosphorylation activation as in the chronic mouse models of epilepsy (Sharma et al., 2018; Alves et al., 2019). Moreover, we have demonstrated the beneficial effects of Fyn and tau reduction/absence on seizures and associated neurobiological changes. Although the majority of the parameters observed in the DKO were similar to either tau KO (more so) or Fyn KO, the robust neuroprotection, suppression of microgliosis, and prevention of polarization of microglia were better achieved in DKO than the single KOs. Further investigations will unravel the mechanisms of such processes. Nonetheless, the new observations presented in this study underscore the role of Fyn, tau, and their interaction as potential therapeutic targets at the early stage of epileptogenesis to prevent or modify the disease onset and its progression.

## Data Availability Statement

The raw data supporting the conclusions of the article will be made available upon request to the corresponding author.

## Ethics Statement

The animal study was reviewed and approved by The University of Iowa Institutional Animal Care and Use Committee.

## Author Contributions

MP, GLi, and SP carried out seizure induction. MP analyzed behavioral seizures, collected samples, performed immunostaining, western blotting, PLA and acquired and analyzed the data, and wrote the manuscript. SP cross-verified the behavioral data analysis. TT and GLe conceived the ideas, secured funding, and supervised the experiments. GLe and TT reviewed and edited the manuscript. All authors contributed to the article and approved the submitted version.

## Conflict of Interest

The authors declare that the research was conducted in the absence of any commercial or financial relationships that could be construed as a potential conflict of interest.
