# Supplementary material for: Fyn-tau Ablation Modifies PTZ-Induced Seizures and Post-seizure Hallmarks of Early Epileptogenesis
Source: Front Cell Neurosci. 2020 Dec 8;14:592374. doi: 10.3389/fncel.2020.592374 (PMC7752812; doi:10.3389/fncel.2020.592374)
Supplement: Supplementary file 1 [file Data_Sheet_1.PDF]

Supplementary Material

Supplementary Figures

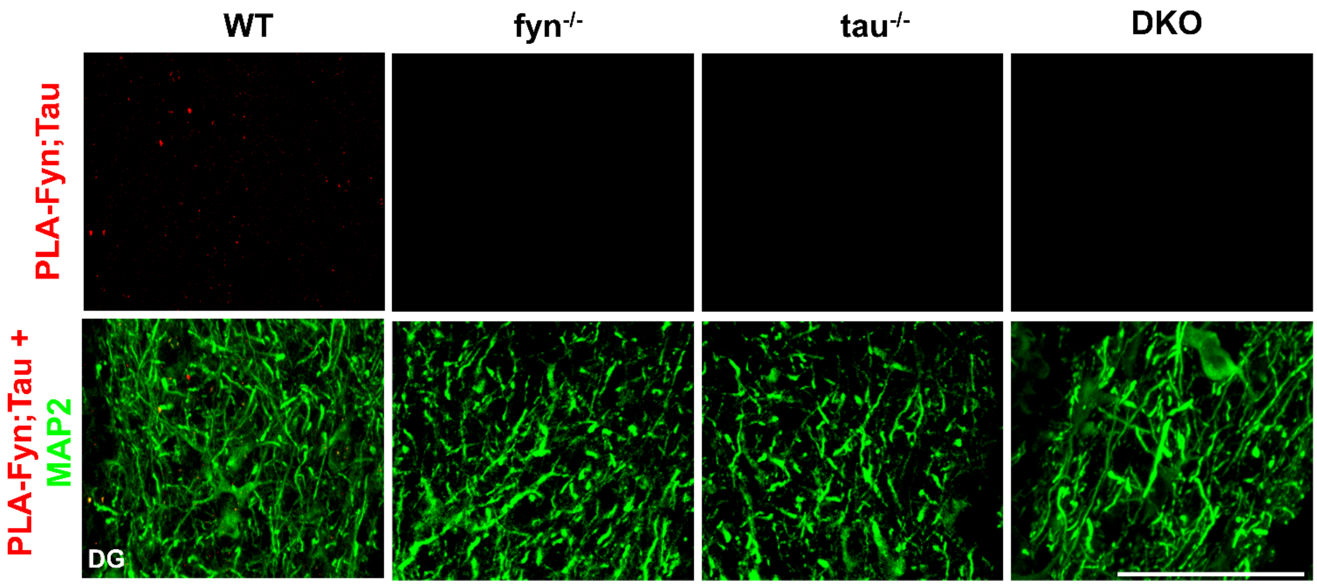

Supplementary Figure 1. Validation of PLA signals with each knockout animal. Scale bar, 100 µm.

Figure 1B

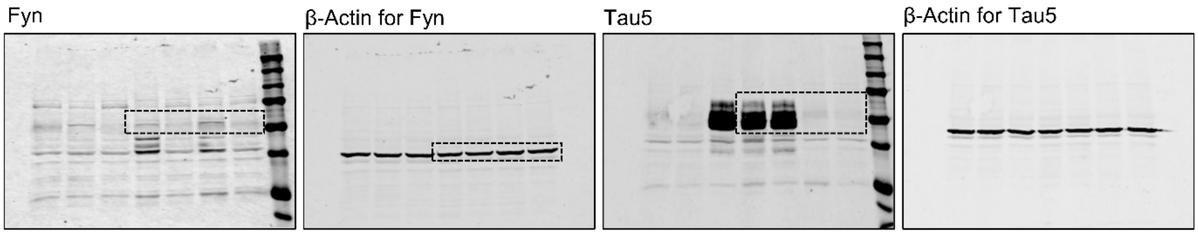

Figure 3A

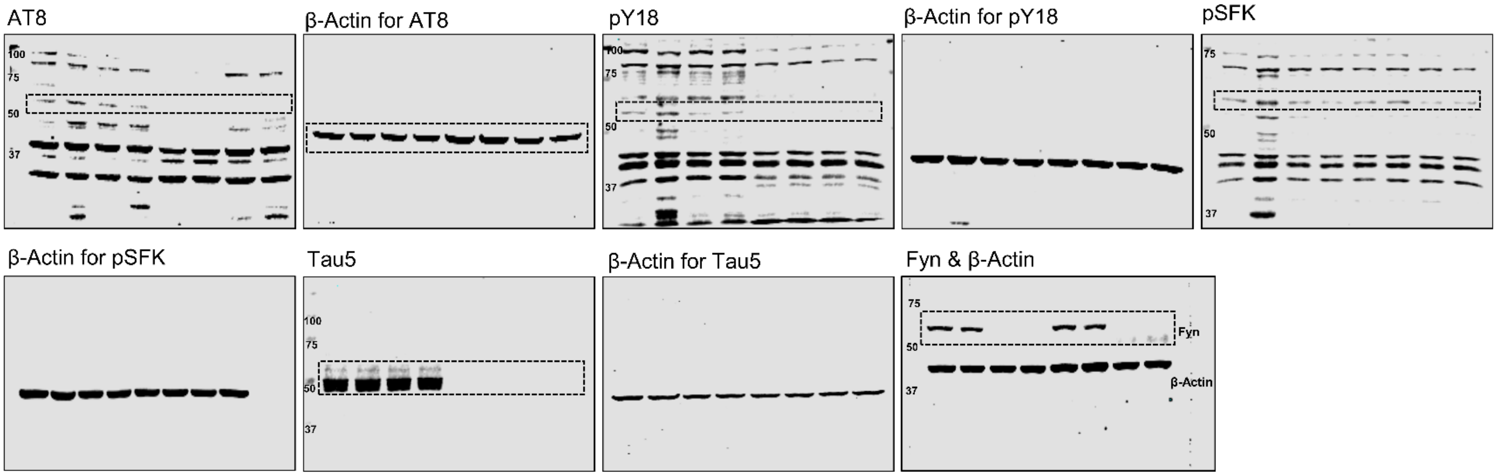

Figure 7C

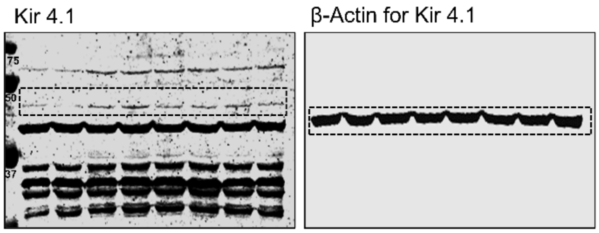

Supplementary Figure 2. Original full western blot image of main manuscript.
